# Inhalable Nanotechnology-Based Drug Delivery Systems for the Treatment of Inflammatory Lung Diseases

**DOI:** 10.3390/pharmaceutics17070893

**Published:** 2025-07-09

**Authors:** Doaa Elsayed Mahmoud, Seyedeh Hanieh Hosseini, Hassaan Anwer Rathore, Alaaldin M. Alkilany, Andreas Heise, Abdelbary Elhissi

**Affiliations:** 1 Department of Pharmaceutical Sciences, College of Pharmacy, QU Health, Qatar University, Doha 2713, Qatar; dm1601604@qu.edu.qa (D.E.M.); hrathore@qu.edu.qa (H.A.R.); alkilany@qu.edu.qa (A.M.A.); 2 Department of Biomedical Sciences, College of Health Sciences, QU Health, Qatar University, Doha 2713, Qatar; sh2401290@student.qu.edu.qa; 3 Department of Chemistry, Royal College of Surgeons in Ireland, University of Medicine and Health Sciences, D02 YN77 Dublin, Ireland; andreasheise@rcsi.ie

**Keywords:** nanoparticles, inhalation devices, drug delivery, inflammatory lung disorders

## Abstract

This review explores recent advancements in inhaled nanoparticle formulations and inhalation devices, with a focus on various types of nanoparticles used for inhalation to treat inflammatory lung diseases and the types of devices used in their delivery. Medical nebulizers have been found to be the most appropriate type of inhalation devices for the pulmonary delivery of nanoparticles, since formulations can be prepared using straightforward techniques, with no need for liquefied propellants as in the case of pressurized metered dose inhalers (pMDIs), or complicated preparation procedures as in the case of dry powder inhalers (DPIs). We demonstrated examples of how formulations should be designed considering the operation mechanism of nebulizers, and how an interplay of factors can affect the aerosol characteristics of nanoparticle formulations. Overall, nanoparticle-based formulations offer promising potential for the treatment of inflammatory lung diseases due to their unique physicochemical properties and ability to provide localized drug delivery in the lung following inhalation.

## 1. Introduction

Inflammation in the lungs originally has a sentinel function against harmful injuries and stimuli through its complex recognition and containment mechanisms. However, uncontrolled or overly responsive inflammatory reactions in the lungs can bring about undesirable complications that can lead to organ dysfunction or tissue damage. This can eventually prompt the development of numerous acute inflammatory lung disorders (AILDs) such as acute respiratory distress syndrome (ARDS)/acute lung injury (ALI), pneumonia, and chronic inflammatory lung disorders (CILDs) such as asthma, chronic obstructive pulmonary disease (COPD), pulmonary fibrosis, and emphysema [1]. Lung inflammatory diseases constitute a huge burden on the healthcare system because they cause substantial morbidities, mortalities, and economic pressure. According to the World Health Organization (WHO), in 2021, COPD and lower respiratory tract infections ranked the fourth and fifth among the leading causes of death worldwide, respectively [2], while asthma was found to affect above 339 million people around the globe. In addition, more than 3 million deaths worldwide every year are ascribed to COPD, representing 6% of the global mortalities [3,4,5,6].

Initially, the inflammation develops secondary to the recognition of external inflammatory stimulus, which provokes the innate immune system to initiate an acute inflammatory response. Such a response can last for hours to days, yet, if the response is excessive or failed to extinguish the inflammation, it will shift into a chronic phase that extends to involve the adaptive immune system, persisting for weeks to years [1]. Inflammatory lung diseases present significant challenges in their management and treatment. Inflammatory responses, if not treated, may lead to perilous complications such as cancer [7]. Despite the presence of well-known therapeutic modalities for inflammatory lung diseases such as oxygen therapy, immunomodulators, and bronchodilators, it remains difficult to ignore their shortcomings, such as the limited efficacy, adverse effects, and poor solubility of some drugs, affecting safety and effectiveness [8,9,10,11]. This necessitated finding alternative, more advanced approaches to circumvent the pitfalls seen with conventional therapies and traditional formulations. From this perspective, the concept of nanoparticle-based therapy has emerged as a promising means for producing targeted drug delivery systems for the treatment of inflammatory lung diseases [12,13,14].

Materials in the nano-size range have unique physicochemical properties that allow them to serve as a viable tool in delivering therapeutic molecules and enhancing their cellular uptake [15,16]. Owing to their small sizes (≤100 nm) [3], they possess high surface area; thus, they are able to enhance the solubility of poorly soluble drugs (i.e., Biopharmaceutics Classification System (BCS) Class II and IV drugs), improving drug absorption and bioavailability [16,17]. This assists in increasing the drug efficacy, reducing adverse effects and improving targetability [17]. The versatile applications of nanoparticle-based formulations and their flexibility for surface and size manipulations have bestowed additional virtues to their properties and recently made them garner significant attention in various fields [18]. To ensure the effective treatment of inflammatory lung diseases, nanoparticle-based formulations including anti-inflammatory drugs should be present in the lung at therapeutic concentrations. Inhalation route is deemed favorable in the case of inflammatory lung diseases, as it contributes to achieving local and optimal drug distribution, deposition, and efficacy [19]. The lung is an appealing site for drug delivery, since it has a large surface area of about 70 m^2^ to 140 m^2^ in an average adult human. The pulmonary route allows for faster transportation of active pharmaceutical ingredients throughout the respiratory epithelium, because of the high blood flow within the pulmonary system [20]. Furthermore, the pulmonary route bypasses the first pass hepatic metabolism; thus, it has much lower levels of metabolic enzymes compared to the gastrointestinal (GI) tract and is a non-invasive, easy method that allows for self-administration of the formulation [20,21]. This review aims to discuss the advancements in inhaled nanoparticle formulations used for the treatment of inflammatory lung diseases and evaluates the suitability of various types of inhalation devices for nanoparticle formulations.

## 2. Lung Barriers and Aerodynamic Considerations When Designing Nanoparticles for Pulmonary Drug Delivery

To ensure the effective pulmonary delivery of nanoparticles, several considerations are required to be discussed. These include the various anatomical and biological barriers confronting the nanoparticles throughout their journey in the respiratory tract, until reaching their intended site. In addition, the aerodynamic considerations are fundamental for engineering nanoparticles with appropriate size, dispersibility, and distribution [22]. This section outlines the key pulmonary barriers affecting the drug delivery of nanoparticles and discusses core aerodynamic considerations for effective pulmonary drug delivery.

### 2.1. Pulmonary Barriers Affecting Drug Delivery of Nanoparticles

**Figure 1:**
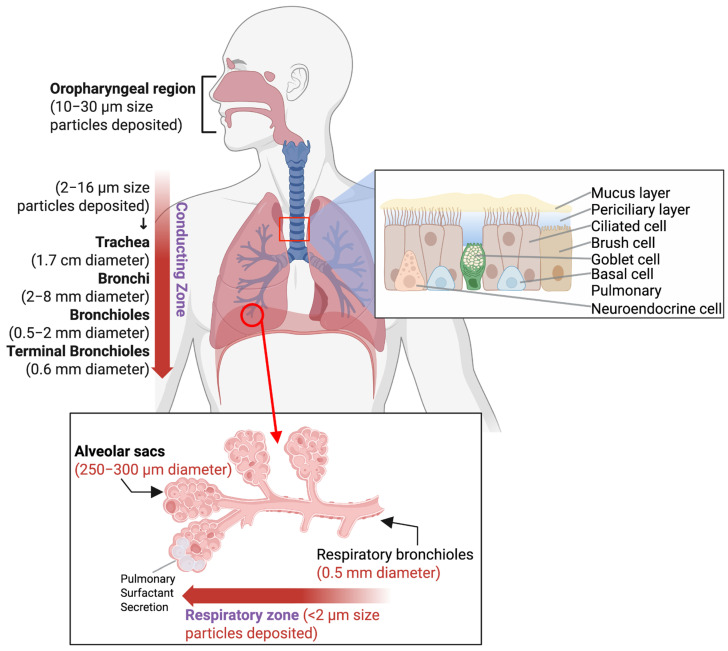
Schematic illustration of the pulmonary barriers confronting the particle during its journey to the respiratory zone in the respiratory system. The airways are lined with different types of cells, each having their own unique function. This involves the epithelial cells of the airways, the goblet cells that produce mucus, the ciliated cells that expel mucus, and the basal cells that have a role in tissue repair and regenerating respiratory epithelium. The diagram also demonstrates the corresponding diameter of each section of the respiratory system and the eligible sizes for particles to deposit. Created in BioRender https://BioRender.com/qlbk9ns (accessed on 2 June 2025).

Given their persistent susceptibility to external pathogens and particles, the lungs possess complex protective barriers that contribute to halting the delivery of nanoparticles and micro-sized particles [23,24]. The complexity in the architecture of the respiratory system makes the mission of delivering effective therapy to the lungs considerably challenging [25]. The barrier system of the lungs consists mainly of four defense mechanisms. The first is a continuous layer of epithelial cells that separates the external environment from the body. The second is the mucociliary clearance, along with the coordinated movement of ciliated cells and the production of mucus [26,27]. It is noteworthy that, in the case of inflammatory lung diseases, these mechanical barriers are notably more robust due to mucus hypersecretion and bronchoconstriction, which engenders a thicker mucus layer, interfering with drug delivery [25]. For instance, George et al. reported that, in diseases such as pneumonia, inflammation leads to filling the alveoli with pus or fluid, negatively affecting their gas exchange function. Furthermore, the physiological changes in asthma due to inflammation causes the airways to narrow (especially the bronchi and bronchioles) and the muscles to constrict, and leads to excessive mucus production, resulting in symptoms like shortness of breath and chest tightness [28,29]. COPD can lead to bronchitis, causing inflammation and bronchi narrowing, in addition to destroying the alveolar wall, resulting in airflow obstruction [29,30]. All these changes can complicate the process of effective nanoparticle delivery. For example, studies have shown that nanoparticles with agglutinated proteins tend to accumulate in inflamed lungs, regardless of their protein composition, zeta potential, and size [31]. The third barrier is the proteins and the antimicrobial peptides present in the lung surfactant, acting as a chemical barrier. Lung surfactant has been shown to influence the fate of inhaled nanoparticles, interacting differently with them based on their various physicochemical properties (e.g., hydrophobicity, particle size, and surface charge) [32,33]. The last one is the miscellaneous immune and non-immune cells that work in coordination to create the immunological barrier [23]. The lung branching structure, considering all its bifurcations, makes it arduous to access the alveolar region after bypassing the upper airways [25] (Figure 1).

Drugs that are not cleared by mucociliary clearance will confront chemical and immunological barriers, such as enzymes, pulmonary surfactants (with a thickness of around 180–890 nm), and immune responses, which can all contribute to the elimination of the drug or the attenuation of its effect [25,34]. These physiological barriers, along with the immunological and chemical barriers, are critical in the protection of the lung from pathogens. However, they constitute an essential obstacle against nano-preparations, which were found to interact with them upon inspiration, resulting in toxicities [35]. Further research is needed to test nanoparticle-based formulations in disease-specific environments and not only in healthy environments, because diseases can further alter the biological responses and affect the safety of such formulations [36]. Indeed, an in-depth understanding of the lung barriers will assist in the design and development of targeted and effective therapies for lung diseases.

### 2.2. Aerodynamic Considerations for Effective Pulmonary Drug Delivery

Achieving effective pulmonary drug delivery with nanoparticles requires careful attention to their aerodynamic behavior within the respiratory tract. There are three primary mechanisms for particle deposition in the lungs: Brownian diffusion, gravity sedimentation, and inertial impaction [37,38]. Ideally, nanoparticles should form inhalable aggregates or be incorporated within carriers with an aerodynamic diameter in the range of 1–5 µm to maximize the chance of deep lung deposition, and be considered “respirable”. Additionally, device parameters must be optimized, and the right design should be selected to preserve the nanoparticles from damage while promoting effective deposition. These considerations are essential for maximizing therapeutic efficiency and minimizing the off-target effects of inhaled nanomedicine. Figure 1 illustrates the respiratory system barriers along with the corresponding diameter of each section of the respiratory system and the eligible sizes for particles to deposit.

## 3. Inhalation Drug Delivery Systems

Pulmonary drug delivery encompasses many types of formulations and aerosol-generating devices [39]. The most current and widely used types will be examined, along with the specific limitations associated with each one. Four devices for pulmonary drug delivery are available on the market: pressurized metered dose inhalers (pMDIs), dry powder inhalers (DPIs), soft mist inhalers (SMIs), and medical nebulizers [40]. Recently, the use of intelligent digital systems has also emerged as a powerful replacement for conventional inhalation devices [41]. The inhalational formulations delivered by these devices are mostly in the form of aerosolized liquid or powder [39].

### 3.1. Pressurized Metered Dose Inhalers (pMDIs)

**Figure 2:**
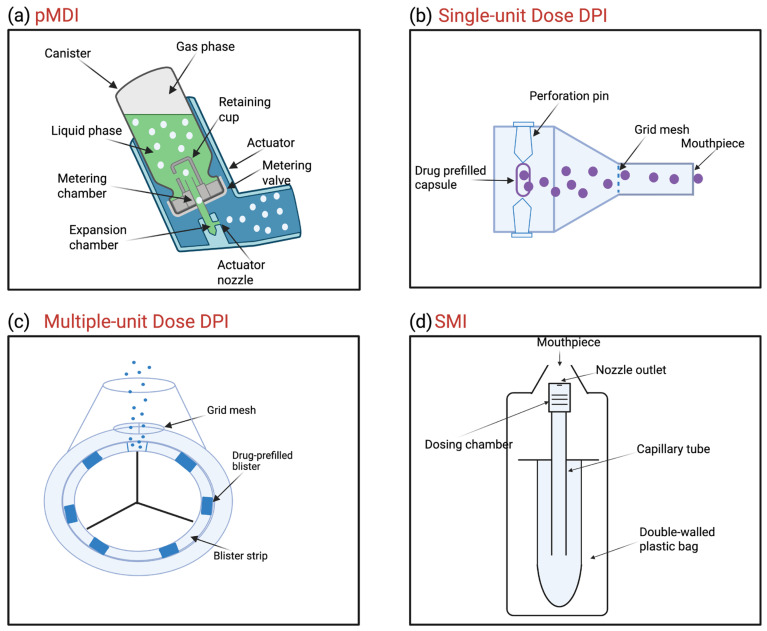
A schematic presentation for the design of (**a**) pressurized metered dose inhaler (pMDI), (**b**) single-unit dose DPI, (**c**) multi-unit dose DPI, and (**d**) soft mist inhaler (SMI). Created in BioRender.

Pressurized metered dose inhalers (pMDIs) are the most commonly used inhalation device [42,43]. A standard pMDI device consists of a canister, retaining cup, metering valve, metering chamber, expansion chamber, actuator, and actuator nozzle [44,45] (Figure 2a). The inner body of the canister is made of inert materials to endure pressure and corrosion that can possibly be caused by the liquefied propellant gas [46]. The propellant, which contains either a drug in solution or a colloidal suspension of a micronized drug, is one of the most important components of pMDIs, as it controls several parameters affecting the delivery and efficiency of the medication. For instance, the propellant evaporation time can affect the dosing because it brings about variations in the physicochemical characteristics and performance of the aerosolized particle (i.e., size distribution, aerodynamic diameter, speed, and oropharyngeal deposition) [47]. pMDIs offer the advantage of providing fixed doses and easy portability [43]. However, lack of coordination during administration of pMDI aerosol often results in oropharyngeal deposition, especially when used by children [48,49]. Moreover, pMDIs have started to employ hydrofluoroalkane (HFA) propellants instead of chlorofluorocarbon (CFC), after CFCs were shown to deplete the ozone layer, potentially exacerbating global warming concerns. However, although HFAs are relatively safer than CFCs, they continue to have high global warming potential, mainly due to the compositions 1,1,1,2,3,3,3-heptafluoropropane (HFC-227ea) and 1,1,1,2-tetrafluoroethane (HFC-134a). Both, in a recent study, were shown to have a global warming potential of 3350 and 1300, respectively [50]. Now more environmentally friendly hydrofluorocarbons (i.e., hydrofluoroolefins; HFOs) have been introduced [51,52]. To support a more environmentally friendly devices for the future, there are several other strategies to minimize the global warming concerns of propellants and make inhalers more sustainable. These include inhaler recycling [53], the switch to inhalers with lower global warming potential, such as DPIs [54], the use of dose counters to avoid misuses or wastes [55], and the use of simpler dose regimes or small-volume pMDIs to minimize propellant release, such as HFA134-based pMDIs [56].

### 3.2. Dry Powder Inhalers (DPIs)

Dry powder inhalers (DPIs; Figure 2b,c) are portable devices containing preparations of micronized dry medication for inhalation, either blended with a carrier or alone. DPIs have more chemical stability and are especially designed to enable the patients to actively inhale the medications for deposition into the lungs [39]. However, the manufacture of this type of inhalation devices is challenging [57]. Therefore, multiple designs of PDIs were proposed; hence, different classifications were provided based on the aerosolization mechanism [58,59]. DPI devices are mainly classified into two groups of either passive (breath-activated) or active (power-driven mechanism) devices [39,43]. The passive DPIs depend mainly on the airflow generated by inhalation as the sole source of power driving the movement and direction of particles from the device to the respiratory tract. In contrast, the active DPIs are supplied with an internal energy source, such as compressed gas, battery, or spring, to generate the aerosol [58]. It has been reported that the limitations of DPIs are mainly due to the need for a high inspiratory flow rate [40]. With a DPI, a drop in pressure by approximately 1 or higher Kilopascal was the threshold above which the delivery of a sufficient dose for inhalation by the patient can be achieved [60]. Furthermore, the powder of the DPIs might exhibit sensitivity to heat and humidity [48], possibly affecting the aerosol performance and pulmonary deposition [61]. DPI devices have multiple designs; thus, they can be for single-dose delivery (Figure 2b) or for multi-dosing purposes (Figure 2c).

### 3.3. Soft Mist Inhalers (SMIs)

Soft mist inhalers (SMIs) are propellant-free metered dose inhalers that release a soft mist of the drug (i.e., slow-moving micro-sized droplets that incorporate the drug). One design of SMIs employs a spring-loaded mechanism as a source of compression power at the base of the device to push the liquid medication through a tiny nozzle (also called a uniblock system). The nozzle creates an angle that allows for the convergence of particles, and hence produces fine slow-moving aerosol droplets with optimized droplet size and velocity [39,62,63,64,65]. A typical soft mist inhaler is composed of a double-walled plastic bag, capillary tube, dosing chamber, nozzle outlet, and a mouthpiece (Figure 2d) [66]. SMIs assist patients in overcoming the hurdles encountered with DPIs and pMDIs through their ability to produce slow-moving aerosols with lower drug deposition in the oropharyngeal region, offering effortless and consistent aerosol delivery [67]. The soft mist produced can last for around 7–10 folds longer than typical aerosols generated from pMDIs, due to the lower velocity of the aerosol particles generated by SMIs [68,69]. This also aids in reducing the amount of effort needed from the patient to maintain synchronization with the device [68,69]. On the other hand, the limitations experienced with SMIs include their relatively higher costs compared to other inhalers, the need for the dose to be loaded into the device, the need for basic assembly and priming, especially if not actively used for over 21 days, and the fact that they are not breath-actuated [48].

### 3.4. Medical Nebulizers

**Figure 3:**
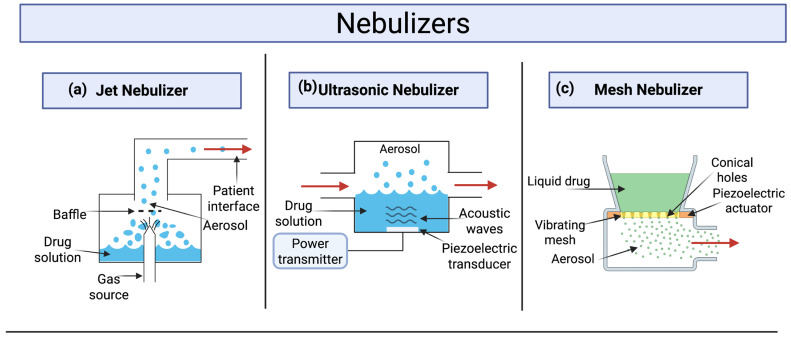
A schematic presentation demonstrating the general design of the three types of nebulizer: (**a**) jet nebulizer, (**b**) ultrasonic nebulizer, and (**c**) mesh nebulizer. Created in BioRender.

Medical nebulizers, simply called nebulizers, are devices intended to deliver the drug to the lower respiratory tract, after converting the drug suspension or solution into a fine mist of micro-sized droplets appropriate for deep lung deposition. Nebulizers are capable of delivering high doses of multiple drugs simultaneously from simply prepared solutions or suspensions, with no need to include a propellant, as in the case of pMDIs, or apply complicated formulation procedures, as in the case of DPIs [39]. Furthermore, nebulizers are suitable for a wider range of patient populations, including elderly, children, those who find it difficult to use pMDIs and DPIs, and critically ill patients [58,70]. Nebulizers can deliver the drug while the patient is breathing normally [39]. In addition, they rely on their own source of power to generate and drive the movement of the aerosolized medicines into the deep lung, and accordingly, they are classified based on their mechanisms of aerosol generation into three main categories: air-jet (pneumatic), mesh, and ultrasonic nebulizers (Figure 3) [43,71].

#### 3.4.1. Air-Jet Nebulizers

Air-jet nebulizers (simply called jet-nebulizers) were the first type of nebulizers introduced to the market. They operate by utilizing a high-velocity gas stream generated by a compressor. The gas passes through a small “venturi” nozzle at the base of the nebulizer cup, converting the liquid medication into atomized droplets (Figure 3a) [72]. Those particles are formed in variant large and small sizes, and this is where the role of the nebulizer baffles emerges. The baffle controls the particle size by acting as a filter that allows small-sized droplets (inhalable secondary aerosol) to pass through to the patient, while larger aerosol droplets (the primary aerosol) collide with the baffles and get returned back into the reservoir for further fragmentation [43].

#### 3.4.2. Ultrasonic Nebulizers

Ultrasonic nebulizers were developed thereafter for the purpose of circumventing some of the limitations seen with the air-jet nebulizers. The noise caused by the air-jet nebulizer is reduced in ultrasonic nebulizers, because ultrasonic nebulizers utilize electrical pulses and high-frequency vibrations by a piezoelectric crystal (1–3 MHz) to generate ultrasound waves that form the aerosols (Figure 3b) [73]. This vibration mechanism lowers the drug leakage risk because of the fact that no source of gas is included in the process of aerosols delivery. Furthermore, ultrasonic nebulizers are more favored for aerosolized therapies due to their greater output capability in comparison to air-jet nebulizers [43]. Despite all of the advantages of ultrasonic nebulizers, studies have shown that they continue to have shortcomings in comparison to jet-nebulizers, such as the high residual volumes, unsuitability for delivering suspensions and viscous solutions, and their unsuitability for the delivery of protein-based formulations due to heat generation, which can degrade heat-labile substances [74,75,76,77].

#### 3.4.3. Vibrating-Mesh Nebulizers

Further improvements in nebulizers have led to the emergence of vibrating-mesh nebulizers (also called mesh nebulizers), in an attempt to produce more efficient nebulization with enhanced aerosol properties. Mesh nebulizers can deliver liquid solutions or suspensions, albeit, in the case of suspensions, the performance and output rate is usually lower [43]. One design of mesh nebulizers relies on the employment of “micropump” technology to generate the aerosol. This is achieved by forcing the liquid medication through a perforated plate or a mesh with multiple apertures, resulting in the generation of a slow-moving cloud of droplets having a narrow size distribution, ensuring maximized deposition in the peripheral airways (Figure 3c). Mesh nebulizers are categorized into two main types: passively vibrating and actively vibrating mesh nebulizers. Passive mesh nebulizers mainly rely on a vibrational element transmitting its vibrations through a horn transducer that pushes the liquid medication through a perforated plate. Here, the mesh vibrations are induced by the horn transducer, not by the mesh itself. Whereas in the actively vibrating mesh nebulizers (e.g., the micropump technology), the perforated plate itself is connected to an electric circuit that causes the vibrations, extruding the liquid through, to generate the aerosol droplets [39,78]. Mesh nebulizers offer shortened nebulization times, silent operation, high aerosol output, great portability, low consumption of power, enablement of dose adjustment, and high lung deposition [78,79]. Most importantly, studies have shown that mesh nebulizers have a small residual volume (from 0.1 mL to 0.5 mL) and high respirable fraction; hence, high drug doses can be effectively delivered, potentially maximizing the therapeutic benefit [39,80]. In a study done by Skaria et al., two jet-nebulizers, namely Respironics Side-Stream^®^ and PARI LC^®^ Plus, were compared with the vibrating-mesh nebulizer Omron U22 in terms of nebulization time, MMAD, particle size distribution, residual volume, and inhaled fraction. Overall results revealed improved performance and substantially decreased drug residual volume (i.e., undelivered fraction) using the mesh nebulizer [81]. Moreover, unlike ultrasonic nebulizers, mesh nebulizers do not heat the medication; thus, they pose a minimal denaturation risk to proteins and can be recommended for heat-sensitive drugs [80,82,83]. However, mesh nebulizers warrant further investigation and improvement, including the problem of orifice clogging, necessitating frequent maintenance and cleaning of the mesh when suspensions or viscous drugs are used [39].

## 4. Inhaled Nanomedicine Formulations in Inflammatory Lung Diseases

**Figure 4:**
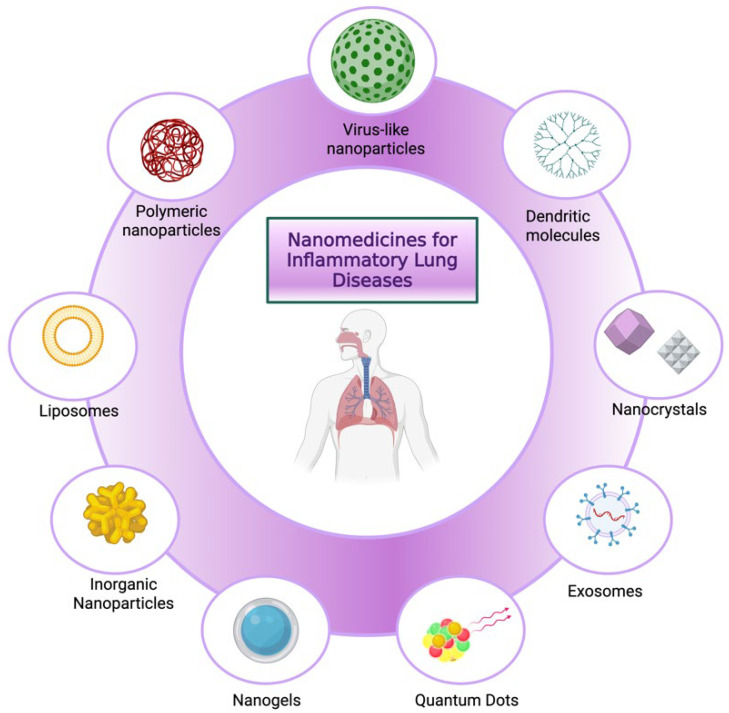
A schematic diagram of nanoparticle formulations investigated for the treatment of inflammatory diseases. Created in BioRender.

“Nanomedicines” is a term that denotes the application of precisely engineered structures with sizes usually ranging from 1 to 100 nm, used for the treatment and diagnosis of diseases [84]. Nanotechnology, in the context of pulmonary drug delivery, has been demonstrated to be a revolutionary means of circumventing lung barriers seen with conventional therapeutics, aiming to attain effective delivery with minimal systemic side effects [85,86]. Nanotechnology-based formulations provide prolonged therapeutic effects and better targetability [33], and besides their excellent localized effect in the lung they can also be used for enhancing drug permeation through the pulmonary tissue, hence delivering the drug through systemic circulation to organs distant from the lung [19]. Recently, an array of nanoparticulate-based formulations have shown improved lung-targeting therapy, such as nanoliposomes, nanopolymersomes, microspheres, and liposomes, especially when surface-modified with different functional groups [87]. In the context of inflammatory lung diseases, nanoparticle-based formulations offer advantages in deep lung delivery due to their superior ability to be incorporated into larger microparticles (1–5 µm) such as the nebulized droplets, which enhances their aerosolization efficiency [88,89]. However, their high surface area can promote aggregation, often requiring the use of stabilizers or formulation strategies to maintain colloidal stability of the nanoparticles [90]. Furthermore, certain inhaled nanoparticle formulations exhibit unpredictable clearance profiles, including lack of biodegradability, which may lead to accumulation and toxicity; thus, using materials that are effective but at the same time biocompatible and biodegradable is essential in the formulation of nanoparticles [33]. Nanoparticle formulations investigated for the delivery via the respiratory system include liposomes, polymer nanoparticles, nanocrystals, exosomes, dendritic macromolecules, inorganic nanoparticles, virus-like particles, and nanogels [33]. Figure 4 is a schematic demonstration of a range of nanoparticle-based systems used in the treatment and diagnosis of inflammatory lung diseases. The subsequent sections elaborate on each type of these nanoparticles, critically reviewing key studies of their delivery to the lung. This is followed by a group of sections evaluating the relationship between inhalation device design and the nanoparticles used for pulmonary delivery.

### 4.1. Liposomes

Liposomes are the most established nanocarrier system used for various drug delivery applications, including pulmonary delivery. Liposomes are spherically shaped phospholipid bilayer vesicles that are amphiphilic in nature and used for drug delivery and targeting. Liposomes, according to their size and morphology, are categorized into small unilamellar vesicles (SUVs; 20–100 nm), large unilamellar vesicles (LUVs; 0.1–1 μm), oligolamellar vesicles (OLVs; 0.1–1 μm), or multilamellar vesicles (MLVs, 0.1–20 μm) [91,92]. Liposomes are capable of entrapping lipophilic and hydrophilic molecules. They also have a composition similar to that of the lung surfactant and cell membranes, rendering them highly biocompatible and biodegradable [33]. As a result, liposomes do not usually stimulate the immune system and are classified as non-toxic materials, making them widely used in food industries, and for cosmetics and pharmaceutical applications [93]. Moreover, the simplicity in their preparation procedure, and their ability to exhibit prolonged retention of the encapsulated drug and the facile uptake by the cells, make them highly promising for pulmonary drug delivery [94,95], with evident successful innovations that reached FDA approval, such as Arikayce^®^ (Table 1). Importantly, drug delivery in liposome nanocarriers via inhalation offers a superior advantage over using the drug in injectable liposome formulations, since the vesicles injected are liable to rapid clearance from the systemic circulation via the reticuloendothelial system [96]. Thus, liposome delivery directly to the lung offers a means for the treatment of pulmonary diseases with no need for mechanisms to protect them against clearance (e.g., PEGylation). Liposomes have demonstrated the ability to encapsulate a wide range of therapeutic molecules, including bronchodilators [97], peptides [98], hormones [99], antibiotics [100], antifungals [101], immunosuppressant drugs [102], anticancer agents [103], and antisense oligonucleotides to target a range of pulmonary diseases [104,105]. Salbutamol is a β1-agonist bronchodilator classified as a BCS III drug that has a short half-life and poor oral bioavailability; thus, inhalation of this drug in liposomal formulation may enhance its localization in the lung and reduce its permeation to the blood circulation, potentially offering enhanced therapeutic outcome [106]. The drug was encapsulated by liposomes made from soya phosphatidylcholine (SPC) and cholesterol using the thin film hydration method. The resultant liposomal formulation demonstrated appropriate aerosol performance and a prolonged drug release, reaching up to 90% over 14 h [94]. Another study was conducted by Leo and co-workers to evaluate the penetration abilities of different liposomal formulations through mucus samples collected from COPD patients. The findings revealed that high mucus penetration occurred within 27 h, with a high safety profile and efficient uptake by the epithelial cells in vitro [107].

### 4.2. Polymeric Nanoparticles

**Figure 5:**
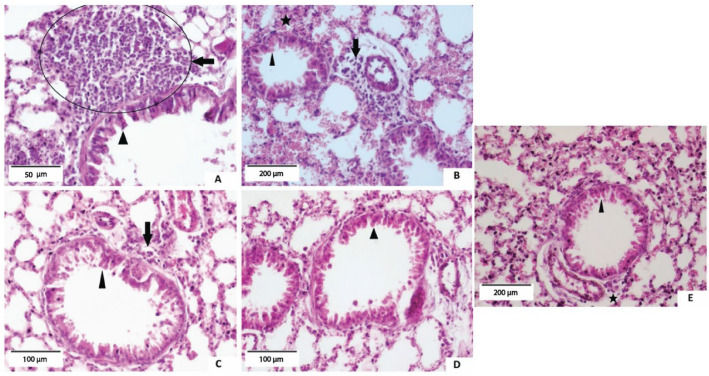
Histopathology of lung sections using hematoxylin and eosin (H&E) stain, after delivery to BALB/c mice using an ultrasonic nebulizer. Arrows indicate inflammatory cell infiltration, arrowheads represent epithelium, and stars depict edema across all images. (**A**) Phosphate buffered saline (control group) depicts edema with severe perivascular and peribronchial inflammatory cell infiltration. (**B**) Dexamethasone positive control group shows moderate perivascular and peribronchial inflammatory cell infiltration with edema. (**C**) Taraxasterol group manifests edema with mild perivascular and peribronchial inflammatory cell infiltration. (**D**) Nano-Taraxcestrol group showcases no perivascular and peribronchial inflammatory cell infiltration with edema. (**E**) PEG-PLGA formulation group shows moderate perivascular and peribronchial inflammatory cell infiltration with edema. The figure was reproduced with permission from [112], Copyright Springer Nature 2024.

Polymeric nanoparticles are biodegradable colloidal systems having a size in the range of 10–200 nm, a range optimal for both site-specific and intravascular targeting and delivery. Polymeric nanoparticles have the capability of deploying specific delivery by virtue of their small hydrodynamic diameters [108,109]. An important aspect in polymeric nanoparticles is that, by encapsulating therapeutic agents, they mitigate their attachment to the mucus, hence facilitating their diffusion through biological membranes [33,110]. In a recent study, dexamethasone-encapsulated polysialic acid and chitosan-based nanoparticles were prepared for treating pulmonary inflammation [111]. In that study, taurine-Vitamin E succinate was used to enhance the electrostatic property of chitosan and polysialic acid, because chitosan is cationic and is able to spontaneously interact with polyanionic polymers to form polyelectrolyte complexes. As a result, acute lung injury treatment and effective targeting against inflammatory macrophages were successfully achieved [111]. Another study focused on the development of taraxasterol-loaded methoxy poly(ethylene glycol)-poly(lactic-co-glycolic acid) (mPEG-PLGA) nanoparticles using the nanoprecipitation method for the treatment of asthma. The new formulation accomplished drug entrapment above 89%, with sustained release over 8 days at physiological body temperature (37 °C). The results of animal studies done in ovalbumin-induced asthma mice models indicated that mPEG-PLGA/taraxasterol acetate formulation was effective at inhibiting the inflammatory markers IgE, IL-13, and IL-4, and demonstrated superiority to the positive control (dexamethasone alone), as shown in the histopathological lung study (Figure 5) [112]. Another recent advancement in the field is the formulation of fluorofenidone-containing spermidine (Spd)-modified PLGA nanoparticles for the treatment of idiopathic lung fibrosis. The resultant product was capable of bypassing reticuloendothelial system and was able to selectively deliver the antifibrotic drug fluorofenidone (AKF). This was evident from the high drug-targeting index of the formulation (Spd-AKF-PLGA NPs) in the lungs in comparison to both controls (AKF solution and AKF-PLGA nanoparticles group). In addition, lung histopathology revealed a noticeable reduction in lung damage in the experimental group in comparison to the control groups [87].

### 4.3. Inorganic Nanoparticles

Inorganic nanoparticles have been employed for different therapeutic and diagnostic purposes due to their distinctive physichochemical characteristics [113]. Inorganic nanoparticles such as those made from gold (AuNPs), silver (AgNPs), and iron oxide (FeNPs) possess unique physical, chemical, optical, and magnetic properties, setting the stage for their efficient utilization in various diagnosis and imaging techniques, such as X-ray computed tomography, fluorescence imaging, and magnetic resonance imaging [114]. From a therapeutic perspective, silver nanoparticles for instance have a well-established antimicrobial activity [115], and recently, the antiviral effects of silver nanoparticles have been reported. The study intended to investigate the behavior of AgNPs in the respiratory tract and evaluate their ability to regulate immune responses secondary to respiratory viral infection in in vivo mice models. The results demonstrated that more lymphocytes, in particular natural killer (NK) cells, were recruited and activated following treatment with AgNPs. Furthermore, AgNPs were shown to be capable of enhancing the ability of the alveolar macrophages to stimulate the production of Interferon gamma (IFN-γ) cytokines, which were reported to markedly induce inflammation after acute exposure in both mice and rat [116]. Iron oxide nanoparticles were also reported to induce inflammation after acute exposure [117,118]. Multiple studies have examined inorganic nanoparticles against respiratory disorders [119,120,121]. For instance, Al Faraj et al. investigated the potential of super-paramagnetic iron oxide nanoparticles (SPIONPs) in lipopolysaccharide-induced COPD mice models. The researchers intended to couple antibodies specific to certain macrophage subpopulations in COPD (M1 and M2) with the SPIONPs and examine their capacity to exert noninvasive, specific, and targeted imaging. The results indicated a successful production of SPIONPs biocompatible for pulmonary administration and effective in detecting M1 and M2 subsets, as evident from the immunohistochemistry analysis. It vividly showed effective localization and targeting of the coupled SPIONPs within the regions of different alveolar macrophage subpopulations, suggesting a promising noninvasive approach for the better and early detection of pulmonary inflammatory diseases [119]. Thus, these studies have demonstrated the capability of inorganic nanoparticles to be used in both the treatment and diagnosis of inflammatory lung diseases. While liposomes have demonstrated advantages in terms of localized lung effects, high compatibility, and superior biodegradability compared to inorganic nanoparticles, the latter have provided an advantage in terms of their potential inherent antimicrobial effect and their possible use in diagnosis of inflammation.

### 4.4. Nanocrystals

Nanocrystals represent another strategy to deliver drugs with poor solubility and low bioavailability. By virtue of their reduced particles size, nanocrystals are able to overcome the pulmonary physiological barriers and attain high drug bioavailability. Furthermore, studies have shown that nanocrystals may offer good safety profiles, high drug loading, and high mucus-penetrating abilities, especially in their rod-shaped forms [20]. A recent study focused on unraveling the solubility and bioavailability challenges associated with the anti-tuberculosis drug clofazimine (CLF) by formulating it into nanocrystals (CLF-NCLs). Using the microfluidizer^®^ technology followed by spray drying, inhalable micro-sized CLF nanoclusters were formulated. The new formulation exhibited a combination of the properties of both nano- and micro-scale particles. The micro-scale particles enabled efficient peripheral lung deposition and provided favorable aerodynamic characteristics, while the embedded nano-scale particles displayed improved the dissolution profile of the poorly water-soluble CLF. Moreover, the inhalable formulation was evaluated in vitro against virulent *Mycobacterium tuberculosis* (MTB) and in vivo using an MTB infected mice model. The findings have demonstrated a significant inhibition of the bacterial cells in the lungs, with a relatively low minimum inhibitory concentration (MIC) [122]. In another recent study, Costabile and co-workers have studied rod-shaped nanocrystals of the C109 drug, which is a potent protein Z inhibitor in *Burkholderia cenocepacia*, the pathogen responsible for Cepacia syndrome (CS), which is a type of pneumonia that occurs in patients with cystic fibrosis [123]. C109 rod-shaped nanocrystals showed smooth penetration through the artificial cystic fibrosis mucus and displayed good inhibitory effects against the pathogen [124]. Thus, overall, nanocrystal formulations demonstrated an activity that depended on their shape and composition, with more studies needed to explore their potential applications in the treatment and diagnosis of inflammatory lung diseases.

### 4.5. Quantum Dots

Quantum dot nanotechnology represents a relatively new area in drug delivery and was first established in the 1980s. Quantum dots are nanocrystals that possess semiconducting properties and unique optical and electronic characteristics, making them potentially good candidates in various technological and scientific domains [125]. Unlike the conventional organic labels, quantum dots have the capacity to produce near-infrared emission, which is sought-after for its high tissue penetration abilities (low abruption and light scattering) [126]. In the field of respiratory disorders, quantum dots have numerous applications, for example, serving as biosensors and drug nanocarriers, in addition to their potential for therapeutic applications and diagnosis (e.g., in biomedical imaging) [126]. Regarding the impact of quantum dots on inflammation, evidence on whether or not they modulate immune responses and inflammation is unclear. Quantum dots may cause damage to the lung epithelial and immune cells and induce inflammatory responses due to several factors, such as their size, surface charge, surface coating, and the presence of toxic materials in their composition [126,127]. A study evaluated the pulmonary biodistribution, clearance, and toxicity of quantum dots in male Sprague-Dawley rats secondary to intratracheal administration. Quantum dots with a cadmium–selenide core were functionalized with different functional groups (carboxyl or amine terminal groups were tested). At different time points, lung inflammation and injury were indicated by lactate dehydrogenase as a marker of cytotoxicity, and albumin as a marker for damage to the air–blood barrier. The results illustrated dose-dependent inflammatory effects peaking at day seven and fourteen post-exposure. The formulations started to destabilize one week post-exposure, and lung deposition as well as clearance were indicated by the rapid phagocytosis by alveolar macrophages in a dose-dependent manner within the first few hours [127]. Similar findings were seen in another study that reported that quantum dots can induce pulmonary inflammation, mainly through manipulating gene expression in lung epithelium and macrophages of mice [128]. In fact, the mechanisms by which quantum dots operate in the respiratory field remain unclear, requiring further research of the factors influencing their operation. Overall, studies do not support the use of quantum dots in pulmonary drug delivery, owing to their significant toxicities.

### 4.6. Exosomes

**Figure 6:**
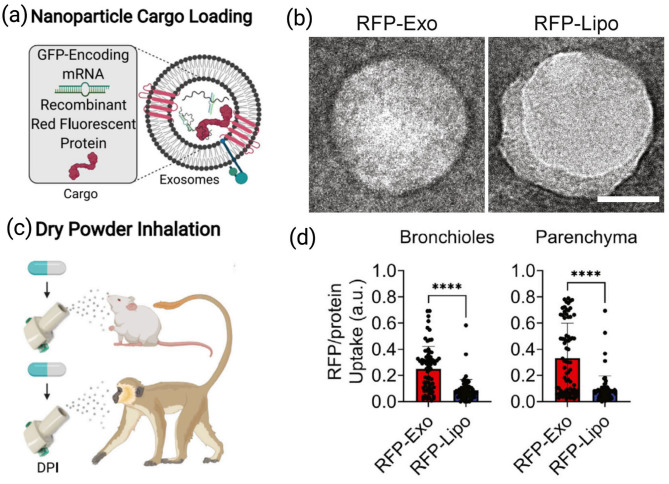
(**a**) Summary of the preparation of Lung-exos nanoparticle formulation. (**b**) TEM images of the nanoparticles of the exosomal (RFP-Exos) and liposomal (RFP-Lipos) formulations; scale bar: 50 nm. (**c**) The delivery of the new formulations using dry powder inhalation. (**d**) Retention and uptake of RFP-Exos formulation in comparison to RFP-Lipos formulation in the lung bronchial and parynchymal regions; **** represents statistical difference. Figure was reproduced with permission from [132], Copyright Elsevier 2022.

Exosomes have gained interest in the field of life sciences and drug delivery. Exosomes are cell-secreted, membrane-bound, nano-sized vesicles that accommodate considerable amounts of proteins, cytokines, and RNA for the treatment of lung diseases [129,130]. Studies have indicated that exosomes may facilitate intracellular communications and play a prominent role as drug carriers owing to their distinctive double membrane structure [33,131]. Popowski et al. have developed a dry powder formulation of lung exosomes (Lung-Exos) that are made to be stable at room temperature. The formulation has been successfully delivered as a dry powder for inhalation, or via nebulization following hydration, using African green monkey (AGM) or mice animal models. Moreover, the distribution of exosomes was superior in the parenchyma and bronchioles compared to conventional liposomes. In vaccine application studies, the lung exosomes loaded with SARS-CoV-2 spike (S) protein encoding mRNA were associated with higher secretory IgA and immunoglobulin G responses compared with the liposomal counterpart (Figure 6) [132]. In another study, curcumin-loaded exosomes modified with RAGE-binding anti-inflammatory peptides (RBP-exo/Cur) were prepared for pulmonary delivery. Both in vitro and in vivo studies confirmed high anti-inflammatory activities and inhibition of inflammatory responses in the lung tissues of mice models with acute lung injury [133]. Collectively, this suggests a promising utilization of these carriers in an array of diagnostic and therapeutic applications.

### 4.7. Other Nanosystems

Several other nanoparticle formulations have been investigated as drug delivery carriers for pulmonary administration to treat inflammatory lung diseases. This includes nanogels, nano-emulsions, dendritic macromolecules, and virus-like particles. For instance, Nasr et al. have used two different lipid nanoemulsions for loading amphotericin B for pulmonary delivery via nebulization. The formulations achieved above 90% drug output, with greater fine particle fraction (FPF) manifested by above 80% deposition in the lower stage of a two-stage impinger [134]. Another trial with nanogels succeeded to produce inhalable quercetin-alginate nanogels with improved bioavailability and solubility of the drug [135]. In addition, dendritic molecules have also been shown to improve the solubility and control the release of the anti-asthma steroid beclometasone dipropionate [136]. Bilosomes are another type of vesicular nanocarrier system that are similar to liposomes but consist of bile salts in their structures. These bilosomes are more stable than traditional liposomal formulations and may offer higher drug encapsulation efficiency. An innovative nanocarrier system based on PEGylated bilosomes (bile bodies) was designed by Zakaria and co-workers, resulting in the successful inhibition of the main protease enzymes of severe acute respiratory syndrome coronavirus-2 [137]. Another type of nanosystem are virus-like nanoparticles. These are nanostructure particles derived from viral proteins but without the viral nucleic acids. During COVID-19, viral-like nanoparticles gained special attention for the delivery of drugs and vaccines. For example, an inhalable COVID-19 vaccine was loaded onto virus-like nanoparticles and lung-derived exosomes. The formulation accomplished an increased retention of cargo in lung parenchyma and mucus-line airways, and in vivo studies revealed reduced inflammatory infiltrates and attenuated severe pneumonia [138].

## 5. Challenges in the Treatment of Inflammatory Lung Diseases

The frequent emergence of new respiratory disorders, the latest of them being COVID-19, highlights a pressing need for more advanced and effective therapies. Nanotechnology is an innovative solution presenting avenues for addressing the limitations seen with conventional therapies and providing enhanced activity against inflammatory pulmonary diseases. However, the knowledge gaps existing between nanoparticle-based formulations and inhalation devices render the actual benefit of all these expeditiously developing nanoparticle-based therapies in the clinical settings questionable. This is manifested by the existence of only one FDA-approved inhalable nano-formulation product as of now (Table 1). In this section, we discuss the main challenges towards the clinical translation of nanoparticles and ensuring their optimal utilization in respiratory delivery research.

### 5.1. Transition from Preclinical Studies to Clinical Trials

Recently, inhalable nanoparticle-based formulations have been broadly explored at the fundamental research level; however, their translation from basic research to clinical application remains notably delayed [88]. A systematic review was done recently to summarize the formulation techniques and preclinical evaluations of inhalable nanoparticle-based formulations for the delivery of anti-tuberculosis drugs. Although a considerable number of preclinical studies have revealed an increased efficacy and reduced adverse effects using inhalable nanoparticles, none has progressed to clinical trials. The reasons the systematic review proposed included the elevated costs of producing and characterizing inhaled regimens and the need for inhalation devices to deliver the formulation, as well as the limited medical adoption [139]. Notably, few clinical trials have been conducted in general on nanoparticle-based therapies targeting inflammatory lung diseases, with Arikayce^®^ being the only one able to make it to the market. The others were either terminated or are still ongoing (Table 1). For example, LUNAR^®^-CFTR lipid nanoparticles are one of the most recent nano-based formulations that was assessed in 2024 for the first time in humans. It is an aerosolized, mutation agnostic, CFTR-mRNA investigational replacement therapy. The new formulation was assessed for its safety, tolerability, and pharmacokinetics in either healthy adults (part 1) or adults with cystic fibrosis (CF) (part 2) in a randomized, single-ascending, placebo controlled clinical trial. To date, part 1 results revealed that the single doses of the formulation were generally safe, with no severe or serious adverse effects. These findings allowed for progression to part 2 of the study, which includes adult patients with CF [140]. Elarekibep (PRS-060/AZD1402) is another example of an inhaled anticalin proteins nanoparticle-based formulation that has progressed to clinical trials. The formulation exhibited a safe and well-tolerated safety profile in phase I, and accordingly, progressed to phase II, in which patients with asthma were enrolled [141]. Table 1 summarizes recent and ongoing clinical trials on inhaled nanoparticles for inflammatory lung diseases.

**Table 1:** Examples of recent inhaled nanoparticles under clinical trials/application for inflammatory lung diseases. From: https://clinicaltrials.gov/ [142]. Accessed on 1 April 2025.

Nanoparticle Formulation	Product Name (Active Pharmaceutical Ingredient)	Disease/Condition	Route of Administration (Device)	Phase (Year of Last Update)	Clinical Trial Identifier/Relevant Reference
Liposome	Arikayce^®^ (Amikacin)	NTM abscessus or MAC lung infections	Inhalation (via nebulization)	Complete—FDA Approved (2018)	NCT01315236/[143]
Lipid	LUNAR^®^-CFTR (ARCT-032)—CFTR mRNA	CF	Inhalation (via nebulization)	Phase II (2025)	NCT06747858/[142]
Nanobodies	LQ036 (IL-4Rα Nbs)	Mild asthma	Inhalation and IV infusion	Phase I (2023)	NCT04993443/[144]
Liposome	Arikayce^®^ (Amikacin)	Pseudomonas infection	Inhalation (via nebulization)	Phase III (2020)	NCT01315678/[145]
Liposome	Arikayce^®^ (Amikacin)	CF	Inhalation (via nebulization)	Phase III (2020)	NCT01316276/[146]
Anticalin proteins	Elarekibep (AZD1402/PRS-060). IL-4Rα antagonist	Mild asthma	Oral inhalation (via nebulization)	Phase I (2020)	NCT03574805/[141]
Anticalin proteins	Elarekibep (AZD1402/PRS-060). IL-4Rα antagonist	Moderate asthma	Oral inhalation (via DPI)	Phase II (2023)	NCT04643158/[147]
Silver nanoparticles	NA	Immune response	Inhalation (via nebulization)	Withdrawn	NCT02408874/[142]
Nanoparticle formulation of Remdesivir	NEUROSIVIR (Remdesivir alone and with NA-831)	COVID-19	Inhalation (via nebulization)	Phase I (2020)	NCT04480333/[142]
Lipid	MRT5005. Codon-optimized CFTR mRNA	Cystic fibrosis	Inhalation (via nebulization)	Phase I/II (2020)	NCT03375047/[148]
Lipid	pGM169/GL67A. CFTR gene–liposome complex	Cystic fibrosis	Inhalation (via nebulization)	Phase II (2015)	NCT01621867/[149]
Liposome	Arikayce^®^/ALIS (Amikacin)	Refractory MAC lung disease	Inhalation (via nebulization)	Phase III (2020)	NCT02344004/[150]
Exosome	Exo-1 and Exo-2 formulations with standard therapy	SARS-CoV-2 Associated Pneumonia	Inhalation (not mentioned)	Phase I/II (2020)	NCT04491240/[151]
Exosome	Exo-1 and Exo-2 formulations with standard therapy	COVID-19 Associated Pneumonia	Inhalation (not mentioned)	Phase II (2020)	NCT04602442/[142]

Abbreviations: FDA: Food and Drug Administration. NA: not applicable. DPI: Dry powder inhaler. Nbs: nanobodies. CF: Cystic fibrosis. CFTR: Cystic Fibrosis transmembrane conductance regulator. mRNA: messenger Ribonucleic Acid. MAC: Mycobacterium Avium Complex. NTM: Nontuberculous mycobacteria. IV: intravenous. ALIS: amikacin liposome inhalation suspension.

### 5.2. Toxicity of the Different Types of Inhalable Nanoparticles

While nanoparticles are an effective means of drug loading and delivery, either locally or systemically, there is accumulating evidence highlighting the toxic effects of several types of nanoparticles on the lungs. This includes the provocation of cytotoxic effects, as well as structural and functional pulmonary changes [152,153]. For instance, nano-Ag, Si_3_N_4_, Fe_2_O_3_, ZrO_2_, Al_2_O_3_, TiO_2_, and chrysotile have been reported to induce toxicity to immortalized human epithelial cells in vitro [154]. Furthermore, it has also been reported that silica nanoparticles, when inhaled, can damage collagen, alveolar structures, and mitochondria [33]. In addition, Wu et al. have summarized the toxic effects of quantum dots on the respiratory system in both in vitro and in vivo studies. The toxicity was evidenced by disordered reactions of immune cells, damage to genetic materials, and reduced cell viability for in vitro tests, whereas in vivo toxicities involved long-term adverse effects, inflammation, lung injuries, and the accumulation of quantum dots in lung tissues [155]. In contrast, biodegradable nanoparticles such as polymeric, lipid-based, protein-based, and biodegradable inorganic nanoparticles all have minimal or no toxicity in vitro and in vivo [152]. Another favorable safety profile was seen with exosomes, which were shown to have only minimal toxicity and high stability and managed to attenuate the side effects of the drug incorporated [156]. Collectively, the toxicity characteristics of nanoparticles vary substantially, given their disparate structural, physical, chemical, optical, and magnetic properties [157]. Furthermore, the potential toxicity of nanoparticles is dependent on particle surface charge, size, and composition; for example, smaller particle size has been reported to exhibit higher biodegradability (i.e., lower toxicity) than larger particles [158,159]. In chronic inflammatory lung diseases, patients with stiffened alveolar walls commonly have higher deposition and preservation of nanoparticles due to reduced clearance and changes in airflow. This is accompanied by a greater transfer of particle composition to systemic circulation, causing potential toxicity to other organs [33]. The overall toxicity of nanoparticles on the respiratory system, as indicated by previous studies, may include genetic changes, fibrosis, respiratory epithelial damage, inflammation, and oxidative stress [160].

Therefore, a special focus on risk mitigation and minimization is crucial in the context of inhaled nanomedicine. Interestingly, Accomasso et al. comprehensively highlighted effective strategies for risk assessment and risk minimization; the most important of which is the safer-by-design approach. This approach is fundamental as it integrates the recognition of potential adverse effects during the early phases of the process of nano-products design, rather than the traditional safety evaluation model. This is because the traditional safety paradigm addresses potential concerns at later stages closer to product and market entry. They also reported the urgent need for the development and validation of novel methods able to predict chronic in vivo outcomes from extrapolated in vitro results, for the purpose of toxicology and safety assessments [36].

### 5.3. Scale-Up and Regulatory Hurdles of Inhalable Nanoparticles

In general, the scalable production and reproducibility of nanoparticles under Good Manufacturing Practice (GMP) conditions are fundamental challenges towards potential clinical translation. Significant batch-to-batch variations, especially in particle size, were observed with various nanoparticle fabrication techniques [161,162]. Scaling up processes were found to bring about additional variabilities [163]. These alterations in the production scale necessitated additional regulations by drug authorities. For instance, despite being approved under the provisions of accelerated approval regulations, Arikayce^®^ underwent a complex process that included extensive product quality reviews and multi-disciplinary reviews (clinical, non-clinical, and statistical), as well as risk assessment and risk mitigation reviews, before receiving the FDA approval (Table 1). Furthermore, the FDA has released an additional guide for the considerations of drug products containing nanomaterials. The guide applies to nano-size engineered drug products, with a special focus on the characterization, testing, control, dissolution and in vitro drug release methods, immunogenicity, stability, and qualification of nanomaterial components. The FDA also stated that additional studies may be required in case the changes in the absorption, distribution, metabolism, and excretion (ADME) of the drug product containing nanomaterials suggest an altered impact on a specific tissue [164].

**Figure 7:**
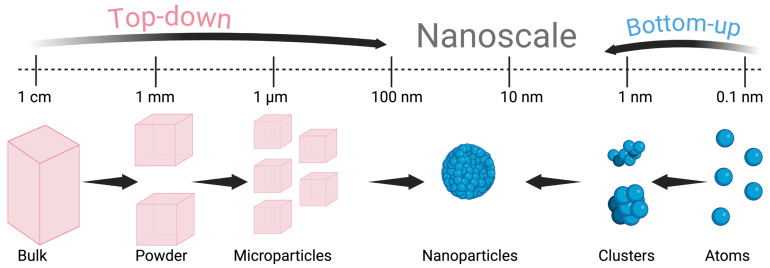
Schematic representation of the principle of top-down and bottom-up techniques used for the synthesis of inorganic nanoparticles. This figure was redrawn using BioRender. with permission from [167], Copyright Elsevier 2024.

The production and scale-up processes of nanomedicines involve a variety of methods, such as the fluidized-bed coating method, spray drying, hot and cold homogenization, nanocrystallization, nanoprecipitation, the milling method, extrusion, supercritical fluid technology, salting out, the ionic gelation technique, and the sonication method [92,165,166]. Scale-up processes usually fall under two main categories: top-down or bottom-up approaches, as demonstrated in Figure 7 [165,167]. The former approach requires the division of bulky masses of the material into smaller particles and is generally done via physical or mechanical means. By contrast, the bottom-up approach usually depends on chemical methods to integrate individual molecules or atoms into larger nanoparticles (i.e., begins with a dissolved molecule until the formation of a precipitate) [167]. It is worth noting that top-down approaches are more popular at the industrial level, because of certain demanding processes associated with the bottom-up techniques, such as the need to remove the traces of the residual solvent [165,168]. Moreover, the adoption of modern strategies such as the Quality-by-Design (QbD) approach, which can employ risk management, analytical, and statistical methodologies in the developments of nanoparticle-based formulations, is highly recommended [161,169]. The QbD approach enables identifying the critical quality attributes (CQAs), the critical material attributes (CMAs), and the critical process parameters (CPPs) of the product [170]. CMAs are the physicochemical, biological, or microbiological attributes that are required to be within an adequate level or limit to ensure appropriate maintenance of the drug’s CQAs across the lifecycle of the product [170]. In the context of inhalable nanoparticles for inflammatory lung diseases, although redispersibility and in vitro aerosol performance are key CQAs, the physicochemical characteristics, such as size, shape, and zeta potential, of the nanoparticles are also important and require optimization for maximum efficacy [161].

### 5.4. Recent Breakthroughs and Their Potential Influence on the Future of Inhalable Nanomedicines

**Figure 8:**
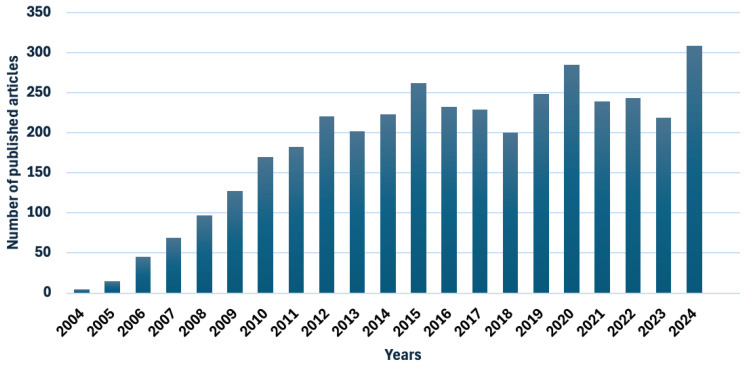
The number of articles published using the word “inhalable nanoparticles” in their title in the PubMed database over the past 20 years. It is evident that this field is gaining more attention with time, especially during the last 15 years.

The field of nanomedicine has witnessed substantial evolution and breakthroughs over the past few decades, driven by the increases in respiratory disease incidences due to rises in the aging population and the intensification of air pollution [171]. The evolution of inhaled nanoparticles was further accelerated with the onset of the COVID-19 pandemic in the years 2020 and 2021 (Figure 8), where novel biodegradable polymers [172] and lipid-nanoparticle-based formulations such as lipid-nanoparticle-based mRNA vaccines gained a special interest [173]. This was followed by the emergence of artificial intelligence (AI) and machine learning and smart inhalation technologies, with their great potential in the field of inhaled nanomedicine. Smart inhalation devices function through tracking, communicating, sensing used information, and providing feedback. The principle of these devices lies in incorporating advanced electronic monitoring systems, mainly through two mechanisms, either self-integration (original integrated devices) or externally (add-on devices). The add-on devices are additional devices attached to a regular traditional inhaler to allow it to perform as a smart inhaler, such as SmartTrack™ and CareTRx™ [174,175]. Whereas the originally integrated ones are devices in which the smart technology is incorporated into them from the beginning, such as those developed by 3M™ Drug Delivery Systems [176]. These digital systems have been employed in various inhalation devices, such as nebulizers, pMDIs, DPIs, and SMIs [41], and their ultimate goal is to minimize medication errors and improve patient compliance [39].

Several trials and studies have been done to evaluate the performance of these devices. For instance, a randomized controlled trial conducted by Chan and co-workers investigated the effect of the use of an inhaler with audiovisual reminders in 220 children with asthma exacerbations. The results illustrated that the use of such electronic monitoring led to a significant enhancement in adherence, and showed promising potential for improving asthma control in those with inadequate control due to poor adherence [175]. Likewise, AI utilization, albeit still considered juvenile, has also shown great promises in aerosol therapy. For example, Chiang et al. employed AI to engineer an AI-base nebulization system able to precisely deliver nebulized aerosol using an intelligent mesh nebulizer. They have used convolutional neural network architecture to analyze the Mel-spectrogram for the breath nasal sounds and activate nebulization during inspiration. The results were outstanding, revealing an increase in drug delivery efficiency by around 250% under different breathing patterns [177]. Therefore, we firmly believe that this technology, together with other advanced techniques such as machine learning, fluid dynamics deposition modeling [178], and multi-omics integration [179] will have substantial contributions in deciphering the interactions between the biological systems and nanoparticles and empowering the field of inhaled nanomedicines.

## 6. Bridging Inhalation Devices and Nanotechnology: Nanoparticle-Device Studies

In spite of the significant importance of establishing compatibility studies between inhalation devices and inhaled nanoparticle-based formulations, there are a limited number of studies highlighting this central issue. Compatibility between formulation and the inhalation device is important with regard to patient safety, consistency of the delivered drug dose and pulmonary deposition, drug stability, and the overall aerosolization performance. A main reason for the limited number of clinically licensed inhalable nanoparticle formulations is their low compatibility with the commercially available inhalation devices [88]. Nanoformulations are delivered to the lungs either in the form of dry microscale powders [161,180,181] or through the nebulization of drug-incorporating nanoparticles within micro-sized aerosol droplets [182,183,184]. Delivery of the nanoformulation as a dry powder or in the form of nebulized aerosol depends on formulation issues, including formulation–device compatibility. The subsequent section intends to highlight the compatibility issues of the available inhalation devices with the various inhalable nanoparticle formulations, particularly those used for the treatment of inflammatory lung diseases.

### 6.1. pMDIs for the Delivery of Nanoparticles

**Figure 9:**
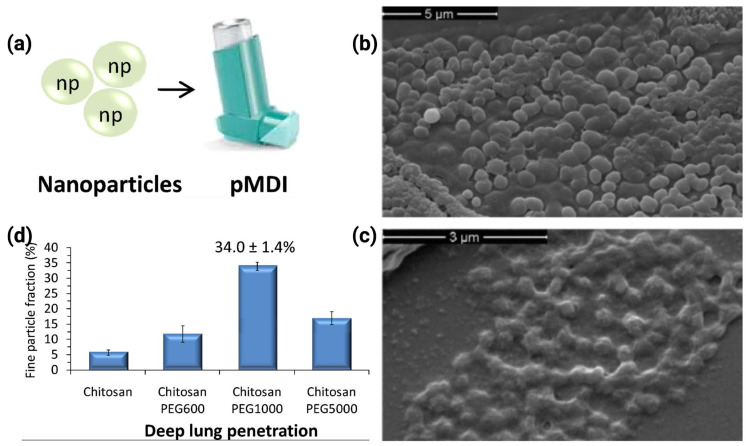
(**a**) Chitosan nanoparticles delivery using a pMDI device. (**b**) Scanning electron microscopy (SEM) images of freeze-dried chitosan–PEG 1000 nanoparticles prior to delivery. (**c**) Scanning electron microscopy (SEM) images of chitosan–PEG 1000 nanoparticles post actuation using the pMDI. (**d**) fine particle fraction percentages of the different chitosan formulations. Figure was reproduced using BioRender with permission from [188]. Copyright Elsevier 2012.

Several previous studies have demonstrated the unsuitability of pMDIs for the pulmonary delivery of nanoparticles [88,185,186]. HFA propellants are the most currently used solvents in pMDIs and offer limited solubilization of many drugs and excipients [51]. Additionally, HFA propellants have a very low boiling point (e.g., around −25 °C) [22]; hence, they will rapidly evaporate upon actuation of the dose [91]. This rapid evaporation together with the high shear forces produced during aerosol generation from the device can confer a destabilizing stress on the nanoparticles, leading to their fragmentation, phase separation, collapse, or aggregation [51]. However, despite the reported low potential of pMDIs for nanoparticle delivery [91,185], some recent investigations have demonstrated that pMDIs are capable of delivering nanoparticles, depending on formulation optimization. For example, Tan and co-workers have shown that lysozyme-containing nanoparticles can be dispersed in HFA propellant. This was achieved through using a freeze-dried formulation incorporating lactose cryoprotectant and lecithin surfactant, resulting in a stable dispersion in HFA134a. The resultant formulation was able to retain as high as 98% of the lysozyme, highlighting the potential of pMDIs to deliver peptide and protein- nanoparticles [187]. Another study by Sharma et al. has emphasized the successful delivery of smooth-surfaced, spherical, crosslinked chitosan–PEG 1000 nanoparticles using a pMDI. The formulation displayed good stability and dispersibility in HFA-227, and a fine particle fraction (FPF) exceeding 30%, demonstrating the potential eligibility for deep lung deposition (Figure 9) [188]. Thus, based on our review, we propose that pMDIs can be suitable for the delivery of nanoparticles, providing that the key parameters affecting their drug deposition and aerodynamic properties are considered. These parameters encompass the physicochemical properties of drugs and excipients (i.e., propellant, cosolvent, surfactant, stabilizing agents, etc.) and the propellant filling method (i.e., pressure filling or cold filling) [189,190,191,192].

### 6.2. DPIs for the Delivery of Inhalable Nanoparticles

DPIs have been involved in the development of various nanoparticle-based formulations, such as polymeric nanoparticles [193], solid lipid nanoparticles [194], nanocomposites [195], and liposomes [196]. In a recent study, nanotechnology has been employed in the development of fixed dose combinations of DPIs of salmeterol xinafoate (SAL) and fluticasone propionate (FP), delivered via tyrosine poly(ester amide)-based nanoparticles as a proposed anti-asthma formulation. The new product demonstrated superior aerodynamic performance with respect to the emitted dose and fine particle fraction (FPF). In vivo studies using mice also revealed promising results manifested by the increased deposition of both drugs in the animal’s lungs compared with the marketed FP and SAL [197]. The general mechanism by which drugs are delivered by DPIs is through the dispersion of the microscale powder containing both the carrier and the drug in the form of an aerosol [198]. The drying methods used for this purpose were spray-drying, freeze-drying, or spray freeze-drying [161]. Freeze-drying (lyophilization) is an optimal selection to maintain the stability of delicate formulations such as vaccine or protein-based nanosystems. However, these techniques make the ingredients more amorphous, necessitating their protection from possible degradation caused by humidity and other inappropriate storage conditions [199]. During spray drying, particle size can be manipulated through controlling the airflow, or by changing the drug or excipients concentrations [199,200]. Spray freeze-drying has been increasingly used to generate particles with large surface areas and enhanced dissolution or dispersion in the aqueous phase [201,202]. Moreover, the protective agents used to stabilize the formulation, such as lactose, can also enhance the aerosol and flow properties of the powder [161]. However, it is important to bear in mind that the inclusion of certain stabilizing agents depends on formulation composition. For example, lactose, which has been proposed as a stabilizing agent and a promoter of the aerosolization properties of powders, can itself interact with protein-based nanoparticles and induce the Maillard reaction, hence causing instability of the formulation [203]. Therefore, it is necessary to consider the compatibility between the ingredients of pulmonary formulations.

### 6.3. Nebulizers for the Delivery of Nanoparticles

So far, the nebulizer is suggested to be the most recommended type of inhalation device for the pulmonary delivery of nanoparticles (Table 2). The use of nebulization for the pulmonary delivery of nanoparticles has gained extensive research interest [91,203]. In a recent study, nebulization of a novel tobramycin dextran-based single-chain polymer nanoparticles (KuDa) was investigated for the treatment of cystic fibrosis. Nebulization has been shown to be appropriate for patients who have difficulty in generating inspiratory efforts due to compromised pulmonary functions [204]. Furthermore, we have previously reported that nebulizers are highly suitable inhalation devices for the delivery of liposomal formulations compared to the other types of inhalation devices, such as pMDIs and DPIs [91]. The ultrasonic nebulizer was found to provide rapid delivery and enhanced drug deposition compared to jet nebulizers. However, ultrasonic nebulizers are not suitable for hypoxic patients due to the low partial oxygen pressure in the respiratory tract secondary to inspiration and the high density of the aerosol generated by the ultrasound waves, indicating that the mechanism of nebulization should consider the patient condition [205]. Importantly, nebulizers can generate aerosols from simple aqueous dispersions of nanoparticles; hence, the complicated formulation strategies used in preparing DPIs or pMDIs are avoided [88,180,206,207]. On the other hand, nebulizers are not free of limitations, such as low portability compared to DPIs and pMDIs, and the need to use large formulation volumes. Furthermore, the intense shearing provided by nebulizers to generate the aerosol may alter the surface properties of the nano-formulation or damage the nanoparticles, leading to several unfavorable outcomes, such as alterations in the drug release profile and loss of the originally encapsulated drug or the carrier coating [208]. Thus, careful assessment of the nebulization effect should be considered.

**Jet Nebulizers:** Although jet nebulizers are the oldest type of nebulizers, they are still the most commonly used devices for the inhalation of suspensions and solutions, because they are relatively inexpensive and can be used for delivering different types of drug formulations, ranging from very simple aqueous solutions to more complicated forms of suspensions, and nanoparticles (e.g., liposomes) [180,209]. Thus, jet nebulizers have been used for a range of nanoparticle types, such as liposomes [210], biodegradable polymeric nanoparticles [183], protein-based nanoparticles [211], polyethyleneimine (PEI) nanoparticles [212], and lipid hybrid nanoparticles [208]. Fu and co-workers have prepared phospholipid-coated nanosuspensions of fluticasone propionate (FP) and investigated their in vitro and in vivo performance. They used an air jet nebulizer for aerosolization studies and analyzed the droplet size and size distribution using laser diffraction and investigated the intratracheal administration of the formulation to the lungs of a rat model. The results conveyed that the phospholipid coating on the nanoparticles was highly desirable, leading to improved lung retention. In addition, jet-nebulization of the nano-FP formulations demonstrated a significant increase in the duration of local drug action compared to a similar microparticle formulation of the drug [213]. Another study evaluated the stability of precirol-based solid lipid nanoparticles (SLPs) during jet-nebulization. Size measurement revealed stability of the nanoparticles through resisting the forces generated during aerosolization. Additionally, post-nebulization, drug nebulization efficiency and aerosol output rate were superior, being around 54% and 150 mg/min, respectively [214].

**Ultrasonic Nebulizers:** Ultrasonic nebulizers have been considered the least suitable type of nebulizer for the delivery of nanocarrier formulations due to various limitations, primarily because ultrasonic nebulizers generate heat during atomization, destabilizing thermolabile formulations like protein and peptide drugs by damaging the chemical structures of biomolecules, with an accompanied change in formulation viscosity and drug concentration in the nebulizer reservoir [88,180,215]. In addition, Ostwald ripening and the agglomeration of particles is also associated with ultrasonic nebulizer, leading to formulation instability [207,216]. Moreover, recent studies showed that mesh nebulizers are greatly replacing ultrasonic nebulizers, since the latter devices, because of the heat generated during their operation, could damage nanoparticles and cause considerable losses of the originally entrapped drug [212]. Despite that, some studies still support the use of ultrasonic nebulizers for the delivery of nanoparticles. For example, a study has compared the three types of nebulizers (i.e., mesh (Aeroneb^®^ Pro), air-jet (PARI LC STAR^®^), and ultrasonic nebulizers (Optineb^®^)) for the delivery of biodegradable nanoparticles in the presence of cryoprotectants. The ultrasonic nebulizer was found to generate aerosols with appropriate characteristics (output rates (≥0.37 g/min), MMAD of 5.3 ± 0.2 to 5.9 ± 0.5 μm, and FPF of around 65%). However, when concentration changes were assessed over 10 min nebulization, the ultrasonic nebulizer was demonstrated to increase the concentration of material compared to the mesh nebulizer, indicating that mesh devices can offer greater uniformity in dose delivery [215].

**Mesh Nebulizers:** Mesh nebulizers have been reported in multiple studies to be equivalent or superior to jet-nebulizers for the delivery of nanoparticle-based formulations [88,217]. They are the newest type of nebulizers with enhanced features able to solve the problems seen with the conventional nebulizers. For instance, they do not cause temperature elevation like ultrasonic nebulizers or reduction like jet devices. Mesh nebulizers are also quiet during operation, while jet nebulizers are noisy because of the employment of compressed gas, which tends to concentrate the drug during operation, resulting in higher residual doses (i.e., undelivered dose fractions) [208,218,219]. On the other hand, the mesh nebulizer requires frequent maintenance and is relatively expensive. A nebulization stress test was conducted using a mesh nebulizer on hybrid nanoarchaeosomes (a novel nanocarrier system derived from archaea-extracted ether lipids) and biogenic silver nanoparticles (BSs). The nebulization studies revealed that the biogenic silver nanoparticles maintained an intact plasmonic peak and size and silver/lipid ratio, and about 90% of the silver was recovered [220]. Recently, the efficiency of mesh versus jet nebulizers in delivering therapeutic nucleic acids encapsulated in polymeric, branched polyethyleneimine-based (b-PEI) nanoparticles was evaluated both in vitro and in vivo. The results indicated the superiority of mesh nebulizers in gene delivery efficiency, as evidenced by the nanoparticle size distribution, higher gene expression level, and the greater concentration of nanoparticles in the lungs. In addition, although both nebulizers demonstrated similar pulmonary deposition levels, the mesh nebulizer outperformed the jet nebulizer in overall gene transfer efficiency in vivo [212]. In another study, a vibrating mesh nebulizer was linked with a whole-body chamber for the aerosolization of mRNA polyplex formulations. The results highlighted the success of the device in generating nanoparticles incorporated into micrometer-sized droplets that are suitable for lung deposition. In addition, the nebulized aerosol succeeded at transfecting around 25% of the lung epithelial cells in vitro and produced significant lung-localized protein expression after a single dose [221]. Thus, the choice of the inhalation device for delivering nanoparticles is dominated by the different characteristics of the device and the formulation.

**Table 2:** A conclusive summary comparing pulmonary inhalation devices with respect to their key features and suitability for nanoparticle formulations.

Inhalation Devices	Key Principle	Advantages	Limitations	Compatible Nanoparticles (Reference)
Jet nebulizers	Utilize compressed air to convert liquid into ‘respirable’ aerosol droplets for passive inhalation by the patient	Compatible with a wide variety of drugs and nano-formulations Suitable for delivery of drugs in simple solutions, suspensions, and nanoparticles Does not generate heat Generally inexpensive	Noisy during operation Bulky in size Risk of drug waste during exhalation High residual doses at the end of nebulization Long time to deliver the needed dose	LNs [222] Biodegradable PNPs [183,223] Protein-based nanoparticles [224] INPs [225] NCs [226] DMs [136] Niosomes [227] SLNPs [214] Exos [228] NEs [229]
Ultrasonic nebulizers	Utilize electrical pulses and vibrations produced by a piezoelectric crystal to create ultrasound waves that can form the aerosols	More portable compared with jet nebulizers Fast delivery of dose Lower risk of drug waste Less noisy	Heat generation causes destabilization of thermolabile materials (e.g., peptide and protein drugs) Changes in formulation viscosity and drug concentration during nebulization (because heat may evaporate the continuous phase) Risk of particle agglomeration because of heating or solvent evaporation	LNs [230,231] INPs [232] NCs [233] NGs [234] Biodegradable PNPs [235] NAs [236]
Mesh nebulizers	Utilize a vibrating mesh or membrane with multiple apertures to create a slow-moving dense cloud of fine aerosol droplets from the liquid medication	High delivery efficiency (i.e., fast) Quiet during operation No temperature change Low residual doses at the end of nebulization Smaller in size compared to jet nebulizers	Requires frequent maintenance and careful use Relatively expensive compared to jet and ultrasonic nebulizers Risk of aperture clogging (especially when using dispersed systems)	LNs [237,238] Biodegradable PNPs [223,239] INPs [240] DMs [136] EVs [131] Exos [241] NGs [242] SDNDs [243] NCs [233] NEs [229]
DPIs	Breath actuated devices that deliver drugs as dry powders into the respiratory airways	Portable and convenient Does not contain a propellant Relatively cost-effective	Dose delivery depends on patients inspiratory force Unsuitable for comatose patients, and elderly patients who need to apply inspiratory force Risk of Maillard reaction with protein-based nanoparticles Risk of powder instability during storage (especially for amorphous powders)	PNPs [244,245] LNs [246,247] EVs [248] Exos [132] Micelles [249] NCs [250] NAMs [251] DMs [252] INPs [253]
pMDIs	Deliver precise doses of medication using propellant-driven aerosol spray	Inexpensive Portable and handy Easy to use Enable precise dose inhalation Multi-dosing device	Require patient consciousness and coordination during inspiration Require pressurized propellants that may pose contamination risks High oropharyngeal deposition is likely, especially if coordinated inhalation is not done properly	PNPs [188] Protein-containing and peptide-containing nanoparticle [187] LNs [254] NEs [255] MEs [256] INPs [257] MCs [258] DMs [252]
SMIs	Propellant-free metered dose inhalers that release a slow-moving soft mist of the drug	Portability is high High drug deposition in the lungs No propellants are included in the formulation Good for patients with limited respiratory coordination Lower oropharyngeal deposition	Relatively expensive Sophisticated process of use Limited information about their compatibility with nanoparticle-based formulations	mRNA encapsulated LNPs [259]

Abbreviations: LNs: Liposomal nanoparticles, PNPs: Polymeric Nanoparticles, INPs: Inorganic Nanoparticles, NCs: Nanocrystals, MCs: microcrystals, EXOs: Exosomes, NGs: Nanogels, NEs: Nano-emulsions, DMs: Dendritic molecules, MEs: Microemulsions, NAs: Nanoaggregates, NAMs: Nano-agglomerates, EVs: extracellular vesicles; SLNPs: Solid-lipid nanoparticles, LNP: Lipid nanoparticles. SDNDs: Solid drug nanoparticle dispersions.

## 7. Conclusions and Future Prospects

The use of nanoparticles has a promising potential in the field of respiratory disorders given the uniform and sustained drug distribution they offer, in addition to their ability to overcome the obstacles posed by the intricate lung anatomy. For each type of device, there is a clear relationship between the characteristics of formulation and the aerosol performance. Therefore, for achieving appropriate deep lung deposition and maximizing the therapeutic benefit of the drug, the right inhalation device should be selected for the right formulation [92]. In spite of the clear inclination of the literature towards mesh nebulizers as the most promising devices for delivering nanoparticle formulations, we uphold the belief that each inhalation device has its own distinct features that can interact differently with different formulations. Hence, there are several factors to be taken into account before determining the compatibility between nanoparticles and the type of inhalation devices. These factors encompass the impact of the inhalation device on the drug physicochemical properties, stability, drug entrapment efficiency, and aerosol deposition in the lungs. Furthermore, the effect of the device on the nano-formulation’s stability, size, agglomeration, concentration, viscosity, fine particle fraction, and most importantly, the ability of the formulation to withstand shearing forces should be considered. Finally, it is noteworthy to mention that we encountered difficulties in locating studies addressing the compatibility of nanoparticles with the inhalation devices. This is because a noticeable number of the available literature focused on the therapeutic outcomes more than on the suitability of the formulation with the inhalation device, although it is crucial to first establish a robust understanding of the mechanism by which the drug is delivered efficiently to the target site.

## Abbreviations

ADME	Absorption, distribution, metabolism, and excretion
AGM	African green monkey
AgNPs	Silver nanoparticles
AI	Artificial intelligence
AILDs	Acute inflammatory lung disorders
ALI	Acute lung injury
ARDS	Acute respiratory distress syndrome
AuNPs	Gold nanoparticles
BALB/c	Bagg Albino c mice
BCS	Biopharmaceutics Classification System
CF	Cystic fibrosis
CFC	Chlorofluorocarbon
CFTR	Cystic fibrosis transmembrane conductance regulator
CILDs	Chronic inflammatory lung disorders
CLF	Clofazimine
CLF-NCLs	Clofazimine nanocrystals
CMAs	Critical material attributes
COPD	Chronic obstructive pulmonary disease
COVID-19	CoronaVirus Disease of 2019
CPPs	Critical process parameters
CQAs	Critical quality attributes
CS	Cepacia syndrome
DMs	Dendritic macromolecules (dendrimers)
DPI	Dry powder inhaler
EVs	Extracellular vesicles
EXOs	Exosomes
FDA	Food and Drug Administration
FeNPs	Iron oxide nanoparticles
FP	Fluticasone propionate
FPF	Fine particle fraction
GMP	Good Manufacturing Practice
H&E stain	Hematoxylin and eosin stain
HFA	Hydrofluoroalkane
HFO	Hydrofluoroolefin
IFN-γ	Interferon gamma
IgE	Immunoglobulin E
IL	Interleukin
INPs	Inorganic Nanoparticles
IV	Intravenous
LNs	Liposomal nanoparticles
Lung-Exos	Lung exosomes
LUVs	Large unilamellar vesicles
MAC	Mycobacterium Avium Complex
MCs	Microcrystals
MEs	Microemulsions
MIC	Minimum inhibitory concentration
MLVs	Multilamellar vesicles
MMAD	Mass median aerodynamic diameter
mPEG	Methoxy poly(ethylene glycol)
mRNA	Messenger Ribonucleic Acid
MTB	Mycobacterium tuberculosis
NAMs	Nano-agglomerates
Nas	Nanoaggregates
Nbs	Nanobodies
NCs	Nanocrystals
NEs	Nano-emulsions
NGs	Nanogels
NTM	Nontuberculous mycobacteria
OLVs	Oligolamellar vesicles
PEG	Polyethylene glycol
PEI	Polyethyleneimine
PLGA	Poly(lactic-co-glycolic) acid
pMDI	Pressurized metered dose inhaler
PNPs	Polymeric nanoparticles
QbD	Quality-by-Design
RBP-exo/Cur	Curcumin-loaded exosomes modified with RAGE-binding anti-inflammatory peptides
SAL	Salmeterol xinafoate
SDND	Solid drug nanoparticle dispersions
SEM	Scanning electron microscopy
SLP	Solid lipid nanoparticle
SMI	Soft mist inhaler
SPC	Soya phosphatidylcholine
SPIONPs	Super-paramagnetic iron oxide nanoparticles
SUVs	Small unilamellar vesicles
TEM	Transmission Electron Microscopy
VLPs	Virus-like particles
WHO	World Health Organization

## References

[1] Brannon E.R., Guevara M.V., Pacifici N.J., Lee J.K., Lewis J.S., Eniola-Adefeso O. (2022). Polymeric particle-based therapies for acute inflammatory diseases. Nat. Rev. Mater..

[2] WHO The Top 10 Causes of Death. https://www.who.int/news-room/fact-sheets/detail/the-top-10-causes-of-death.

[3] Gulati N., Chellappan D.K., MacLoughlin R., Dua K., Dureja H. (2021). Inhaled nano-based therapeutics for inflammatory lung diseases: Recent advances and future prospects. Life Sci..

[4] Stern J., Pier J., Litonjua A.A. (2020). Asthma epidemiology and risk factors. Seminars in Immunopathology.

[5] Bollmeier S.G., Hartmann A.P. (2020). Management of chronic obstructive pulmonary disease: A review focusing on exacerbations. Am. J. Health-Syst. Pharm..

[6] Smalley K.R., Aufegger L., Flott K., Mayer E.K., Darzi A. (2021). Can self-management programmes change healthcare utilisation in COPD?: A systematic review and framework analysis. Patient Educ. Couns..

[7] Gomes M., Teixeira A.L., Coelho A., Araújo A., Medeiros R. (2014). The role of inflammation in lung cancer. Adv. Exp. Med. Biol..

[8] Wang J., Wang P., Shao Y., He D. (2023). Advancing Treatment Strategies: A Comprehensive Review of Drug Delivery Innovations for Chronic Inflammatory Respiratory Diseases. Pharmaceutics.

[9] Christenson S.A., Smith B.M., Bafadhel M., Putcha N. (2022). Chronic obstructive pulmonary disease. Lancet.

[10] Martinez F.J., Collard H.R., Pardo A., Raghu G., Richeldi L., Selman M., Swigris J.J., Taniguchi H., Wells A.U. (2017). Idiopathic pulmonary fibrosis. Nat. Rev. Dis. Primers.

[11] Miller R.L., Grayson M.H., Strothman K. (2021). Advances in asthma: New understandings of asthma’s natural history, risk factors, underlying mechanisms, and clinical management. J. Allergy Clin. Immunol..

[12] Porsbjerg C.M., Sverrild A., Lloyd C.M., Menzies-Gow A.N., Bel E.H. (2020). Anti-alarmins in asthma: Targeting the airway epithelium with next-generation biologics. Eur. Respir. J..

[13] Brightling C., Greening N. (2019). Airway inflammation in COPD: Progress to precision medicine. Eur. Respir. J..

[14] Wang J., Hu K., Cai X., Yang B., He Q., Wang J., Weng Q. (2022). Targeting PI3K/AKT signaling for treatment of idiopathic pulmonary fibrosis. Acta Pharm. Sin. B.

[15] Cheng W.L., Tan S.J., Campolongo M.J., Hartman M.R., Kahn J.S., Luo D., Andrews D.L., Scholes G.D., Wiederrecht G.P. (2011). 3.03—Bio-Mediated Assembly of Ordered Nanoparticle Superstructures. Comprehensive Nanoscience and Technology.

[16] Van Eerdenbrugh B., Vermant J., Martens J.A., Froyen L., Humbeeck J.V., Van den Mooter G., Augustijns P. (2010). Solubility Increases Associated with Crystalline Drug Nanoparticles: Methodologies and Significance. Mol. Pharm..

[17] Liu Y., Liang Y., Yuhong J., Xin P., Han J.L., Du Y., Yu X., Zhu R., Zhang M., Chen W. (2024). Advances in Nanotechnology for Enhancing the Solubility and Bioavailability of Poorly Soluble Drugs. Drug Des. Devel. Ther..

[18] Sanità G., Carrese B., Lamberti A. (2020). Nanoparticle Surface Functionalization: How to Improve Biocompatibility and Cellular Internalization. Front. Mol. Biosci..

[19] Kaur G., Narang R.K., Rath G., Goyal A.K. (2012). Advances in Pulmonary Delivery of Nanoparticles. Artif. Cells Blood Substit. Biotechnol..

[20] Yue P., Zhou W., Huang G., Lei F., Chen Y., Ma Z., Chen L., Yang M. (2022). Nanocrystals based pulmonary inhalation delivery system: Advance and challenge. Drug Deliv..

[21] van Rijt S.H., Bein T., Meiners S. (2014). Medical nanoparticles for next generation drug delivery to the lungs. Eur. Respir. J..

[22] Myrdal P.B., Sheth P., Stein S.W. (2014). Advances in metered dose inhaler technology: Formulation development. AAPS PharmSciTech.

[23] Kageyama T., Ito T., Tanaka S., Nakajima H. (2024). Physiological and immunological barriers in the lung. Semin. Immunopathol..

[24] Murgia X., de Souza Carvalho C., Lehr C.-M. (2014). Overcoming the pulmonary barrier: New insights to improve the efficiency of inhaled therapeutics. Eur. J. Nanomed..

[25] García-Fernández A., Sancenón F., Martínez-Máñez R. (2021). Mesoporous silica nanoparticles for pulmonary drug delivery. Adv Drug Deliv. Rev..

[26] Munkholm M., Mortensen J. (2014). Mucociliary clearance: Pathophysiological aspects. Clin. Physiol. Funct. Imaging.

[27] Ganesan S., Comstock A.T., Sajjan U.S. (2013). Barrier function of airway tract epithelium. Tissue Barriers.

[28] Shukla S.D., Swaroop Vanka K., Chavelier A., Shastri M.D., Tambuwala M.M., Bakshi H.A., Pabreja K., Mahmood M.Q., O’Toole R.F., Dua K., Hansbro P.M., Wadhwa R., Haghi M., Pont L.G., Williams K.A. (2020). Chapter 1—Chronic respiratory diseases: An introduction and need for novel drug delivery approaches. Targeting Chronic Inflammatory Lung Diseases Using Advanced Drug Delivery Systems.

[29] George M., Boukherroub R., Sanyal A., Szunerits S. (2025). Treatment of lung diseases via nanoparticles and nanorobots: Are these viable alternatives to overcome current treatments?. Mater. Today Bio..

[30] Adeloye D., Chua S., Lee C., Basquill C., Papana A., Theodoratou E., Nair H., Gasevic D., Sridhar D., Campbell H. (2015). Global and regional estimates of COPD prevalence: Systematic review and meta-analysis. J. Glob. Health.

[31] Kim J., Sahay G. (2022). Nanomedicine hitchhikes on neutrophils to the inflamed lung. Nat. Nanotechnol..

[32] Xu Y., Parra-Ortiz E., Wan F., Cañadas O., Garcia-Alvarez B., Thakur A., Franzyk H., Pérez-Gil J., Malmsten M., Foged C. (2023). Insights into the mechanisms of interaction between inhalable lipid-polymer hybrid nanoparticles and pulmonary surfactant. J Colloid Interface Sci..

[33] Feng X., Shi Y., Zhang Y., Lei F., Ren R., Tang X. (2024). Opportunities and Challenges for Inhalable Nanomedicine Formulations in Respiratory Diseases: A Review. Int. J. Nanomed..

[34] Patton J.S., Brain J.D., Davies L.A., Fiegel J., Gumbleton M., Kim K.-J., Sakagami M., Vanbever R., Ehrhardt C. (2010). The particle has landed—Characterizing the fate of inhaled pharmaceuticals. J. Aerosol Med. Pulm. Drug Deliv..

[35] Raine R.I. (2003). Technology in respiratory medicine. Contin. Med. Educ..

[36] Accomasso L., Cristallini C., Giachino C. (2018). Risk Assessment and Risk Minimization in Nanomedicine: A Need for Predictive, Alternative, and 3Rs Strategies. Front. Pharmacol..

[37] Rogueda P.G., Traini D. (2007). The nanoscale in pulmonary delivery. Part 1: Deposition, fate, toxicology and effects. Expert Opin. Drug Deliv..

[38] Wei S., Xie J., Luo Y., Ma Y., Tang S., Yue P., Yang M. (2018). Hyaluronic acid based nanocrystals hydrogels for enhanced topical delivery of drug: A case study. Carbohydr. Polym..

[39] Wang B., Wang L., Yang Q., Zhang Y., Qinglai T., Yang X., Xiao Z., Lei L., Li S. (2024). Pulmonary inhalation for disease treatment: Basic research and clinical translations. Mater. Today Bio.

[40] Banat H., Ambrus R., Csóka I. (2023). Drug combinations for inhalation: Current products and future development addressing disease control and patient compliance. Int. J. Pharm..

[41] Xiroudaki S., Schoubben A., Giovagnoli S., Rekkas D.M. (2021). Dry powder inhalers in the digitalization era: Current status and future perspectives. Pharmaceutics.

[42] Raju S., Suryawanshi S. (2014). Use of pressurized metered dose inhalers in patients with chronic obstructive pulmonary disease: Review of evidence. Expert Rev. Clin. Pharmacol..

[43] Chandel A., Goyal A.K., Ghosh G., Rath G. (2019). Recent advances in aerosolised drug delivery. Biomed. Pharmacother..

[44] Vaswani S.K., Creticos P.S. (1998). Metered Dost Inhaler: Past, Present, and Future. Ann. Allergy Asthma Immunol..

[45] Hess D.R. (2008). Aerosol delivery devices in the treatment of asthma. Respir. Care.

[46] Smyth H.D. (2003). The influence of formulation variables on the performance of alternative propellant-driven metered dose inhalers. Adv. Drug Deliv. Rev..

[47] Legh-Land V., Haddrell A.E., Lewis D., Murnane D., Reid J.P. (2021). Water Uptake by Evaporating pMDI Aerosol Prior to Inhalation Affects Both Regional and Total Deposition in the Respiratory System. Pharmaceutics.

[48] Usmani O.S. (2019). Choosing the right inhaler for your asthma or COPD patient. Ther. Clin. Risk Manag..

[49] Newman S.P., Pavia D., Moren F., Sheahan N.F., Clarke S.W. (1981). Deposition of pressurised aerosols in the human respiratory tract. Thorax.

[50] Urrutia-Pereira M., Chong-Neto H.J., Winders T.A., Solé D. (2023). Environmental impact of inhaler devices on respiratory care: A narrative review. J. Bras. Pneumol..

[51] Zhou Q.T., Tang P., Leung S.S.Y., Chan J.G.Y., Chan H.-K. (2014). Emerging inhalation aerosol devices and strategies: Where are we headed?. Adv. Drug Deliv. Rev..

[52] Tewari S.G., Bell J.P., Budgen N., Platz S., Gibbs M., Newham P., Kimko H. (2023). Pressurized metered-dose inhalers using next-generation propellant HFO-1234ze (E) deposit negligible amounts of trifluoracetic acid in the environment. Front. Environ. Sci..

[53] Murphy A., Howlett D., Gowson A., Lewis H. (2023). Understanding the feasibility and environmental effectiveness of a pilot postal inhaler recovery and recycling scheme. npj Prim. Care Respir. Med..

[54] Wilkinson A.J., Braggins R., Steinbach I., Smith J. (2019). Costs of switching to low global warming potential inhalers. An economic and carbon footprint analysis of NHS prescription data in England. BMJ Open.

[55] Tan S.Y., Hoon M., Tan Y.H., Teo A.H., Cheng Z.R., Teoh O.H. (2021). 425 Ensuring safe use of pressurised metered dose inhalers without a built-in dose counter. BMJ Paediatr. Open.

[56] Wilkinson A.J.K., Anderson G. (2020). Sustainability in Inhaled Drug Delivery. Pharm. Med..

[57] Sanchis J., Corrigan C., Levy M.L., Viejo J.L. (2013). Inhaler devices–from theory to practice. Respir. Med..

[58] Ibrahim M., Verma R., Garcia-Contreras L. (2015). Inhalation drug delivery devices: Technology update. Med. Devices Evid. Res..

[59] Ye Y., Ma Y., Zhu J. (2022). The future of dry powder inhaled therapy: Promising or discouraging for systemic disorders?. Int. J. Pharm..

[60] Clark A.R., Weers J.G., Dhand R. (2020). The Confusing World of Dry Powder Inhalers: It Is All About Inspiratory Pressures, Not Inspiratory Flow Rates. J. Aerosol. Med. Pulm Drug Deliv..

[61] Janson C., Lööf T., Telg G., Stratelis G., Nilsson F. (2016). Difference in resistance to humidity between commonly used dry powder inhalers: An in vitro study. NPJ Prim. Care Respir. Med..

[62] Perriello E.A., Sobieraj D.M. (2016). The Respimat Soft Mist Inhaler, a Novel Inhaled Drug Delivery Device. Conn. Med..

[63] Sadeghi T., Fatehi P., Pakzad L. (2024). Effect of Nasal Inhalation on Drug Particle Deposition and Size Distribution in the Upper Airway: With Soft Mist Inhalers. Ann. Biomed. Eng..

[64] Smith G., Hiller C., Mazumder M., Bone R. (1980). Aerodynamic size distribution of cromolyn sodium at ambient and airway humidity. Am. Rev. Respir. Dis..

[65] Wachtel H., Kattenbeck S., Dunne S., Disse B. (2017). The Respimat^®^ development story: Patient-centered innovation. Pulm. Ther..

[66] Carrigy N.B., Chang R.Y., Leung S.S., Harrison M., Petrova Z., Pope W.H., Hatfull G.F., Britton W.J., Chan H.-K., Sauvageau D. (2017). Anti-tuberculosis bacteriophage D29 delivery with a vibrating mesh nebulizer, jet nebulizer, and soft mist inhaler. Pharm. Res..

[67] Iwanaga T., Tohda Y., Nakamura S., Suga Y. (2019). The Respimat^®^ soft mist inhaler: Implications of drug delivery characteristics for patients. Clin. Drug Investig..

[68] Anderson P. (2006). Use of Respimat Soft Mist inhaler in COPD patients. Int. J. Chron. Obs. Pulmon. Dis..

[69] Gumani D., Newmarch W., Puopolo A., Casserly B. (2016). Inhaler technology. Int. J. Respir. Pulm. Med..

[70] Khairnar S.V., Jain D.D., Tambe S.M., Chavan Y.R., Amin P.D. (2022). Nebulizer systems: A new frontier for therapeutics and targeted delivery. Ther. Deliv..

[71] Fink J.B., Stapleton K.W. (2024). Nebulizers. J. Aerosol Med. Pulm. Drug Deliv..

[72] Khan I., Elhissi A., Shah M., Alhnan M.A., Ahmed W., Davim J.P. (2013). 9—Liposome-based carrier systems and devices used for pulmonary drug delivery. Biomaterials and Medical Tribology.

[73] Arı A. (2014). Jet, Ultrasonic, and Mesh Nebulizers: An Evaluation of Nebulizers for Better Clinical Outcomes. Eurasian J. Pulmonol..

[74] Ari A., Fink J.B. (2011). Guidelines for aerosol devices in infants, children and adults: Which to choose, why and how to achieve effective aerosol therapy. Expert Rev. Respir. Med..

[75] Ari A., Restrepo R.D. (2012). Aerosol delivery device selection for spontaneously breathing patients: 2012. Respir. Care.

[76] Taylor K.M., McCallion O.N. (1997). Ultrasonic nebulisers for pulmonary drug delivery. Int. J. Pharm..

[77] Watts A.B., McConville J.T., Williams III R.O. (2008). Current therapies and technological advances in aqueous aerosol drug delivery. Drug Dev. Ind. Pharm..

[78] Hickey A.J. (2020). Emerging trends in inhaled drug delivery. Adv. Drug Deliv. Rev..

[79] Vecellio L., De Gersem R., Le Guellec S., Reychler G., Pitance L., Le Pennec D., Diot P., Chantrel G., Bonfils P., Jamar F. (2011). Deposition of aerosols delivered by nasal route with jet and mesh nebulizers. Int. J. Pharm..

[80] Dhand R. (2017). How should aerosols be delivered during invasive mechanical ventilation?. Respir. Care.

[81] Skaria S., Smaldone G.C. (2010). Omron NE U22: Comparison between vibrating mesh and jet nebulizer. J. Aerosol Med. Pulm. Drug Deliv..

[82] Johnson J.C., Waldrep J.C., Guo J., Dhand R. (2008). Aerosol delivery of recombinant human DNase I: In vitro comparison of a vibrating-mesh nebulizer with a jet nebulizer. Respir. Care.

[83] Ruickbie S., Hall A., Ball J. (2011). Therapeutic aerosols in mechanically ventilated patients. Annu. Update Intensive Care Emerg. Med..

[84] Paszko E., Ehrhardt C., Senge M.O., Kelleher D.P., Reynolds J.V. (2011). Nanodrug applications in photodynamic therapy. Photodiagnosis Photodyn. Ther..

[85] Fu Q., Liu Y., Peng C., Muluh T.A., Anayyat U., Liang L. (2025). Recent advancement in inhaled nano-drug delivery for pulmonary, nasal, and nose-to-brain diseases. Curr. Drug Deliv..

[86] Lim Y.H., Tiemann K.M., Hunstad D.A., Elsabahy M., Wooley K.L. (2016). Polymeric nanoparticles in development for treatment of pulmonary infectious diseases. Wiley Interdiscip. Rev. Nanomed. Nanobiotechnol..

[87] Tang J., Li J., Li G., Zhang H., Wang L., Li D., Ding J. (2017). Spermidine-mediated poly (lactic-co-glycolic acid) nanoparticles containing fluorofenidone for the treatment of idiopathic pulmonary fibrosis. Int. J. Nanomed..

[88] Peng S., Wang W., Zhang R., Wu C., Pan X., Huang Z. (2024). Nano-Formulations for Pulmonary Delivery: Past, Present, and Future Perspectives. Pharmaceutics.

[89] Chiang P.-C., Alsup J.W., Lai Y., Hu Y., Heyde B.R., Tung D. (2009). Evaluation of aerosol delivery of nanosuspension for pre-clinical pulmonary drug delivery. Nanoscale Res. Lett..

[90] Ibrahim H.M., Awad M., Al-Farraj A.S., Al-Turki A.M. (2020). Stability and Dynamic Aggregation of Bare and Stabilized Zero-Valent Iron Nanoparticles under Variable Solution Chemistry. Nanomaterials.

[91] Elhissi A. (2017). Liposomes for pulmonary drug delivery: The role of formulation and inhalation device design. Curr. Pharm. Des..

[92] Elhissi A., Elkhalifa D., Khan I., Ahmed W. (2025). Proliposomes: A Manufacturing Technology of Liposomes for Pulmonary Drug Delivery.

[93] Dymek M., Sikora E. (2022). Liposomes as biocompatible and smart delivery systems—The current state. Adv. Colloid Interface Sci..

[94] Honmane S., Hajare A., More H., Osmani R.A.M., Salunkhe S. (2019). Lung delivery of nanoliposomal salbutamol sulfate dry powder inhalation for facilitated asthma therapy. J. Liposome Res..

[95] Leong E.W.X., Ge R. (2022). Lipid Nanoparticles as Delivery Vehicles for Inhaled Therapeutics. Biomedicines.

[96] Weber S., Zimmer A., Pardeike J. (2014). Solid lipid nanoparticles (SLN) and nanostructured lipid carriers (NLC) for pulmonary application: A review of the state of the art. Eur. J. Pharm. Biopharm..

[97] McCalden T.A., Radhakrishnan R. (1991). A comparative study of the bronchodilator effect and duration of action of liposome encapsulated beta-2 adrenergic agonists in the guinea-pig. Pulm. Pharmacol..

[98] Chellappan D.K., Prasher P., Saravanan V., Yee V.S.V., Chi W.C.W., Wong J.W., Wong J.K., Wong J.T., Wan W., Chellian J. (2022). Protein and peptide delivery to lungs by using advanced targeted drug delivery. Chem.-Biol. Interact..

[99] Gallez A., Palazzo C., Blacher S., Tskitishvili E., Noël A., Foidart J.-M., Evrard B., Pequeux C., Piel G. (2020). Liposomes and drug-in-cyclodextrin-in-liposomes formulations encapsulating 17β-estradiol: An innovative drug delivery system that prevents the activation of the membrane-initiated steroid signaling (MISS) of estrogen receptor α. Int. J. Pharm..

[100] Cipolla D., Blanchard J., Gonda I. (2016). Development of liposomal ciprofloxacin to treat lung infections. Pharmaceutics.

[101] Lowry C., Marty F., Vargas S., Lee J., Fiumara K., Deykin A., Baden L. (2007). Safety of aerosolized liposomal versus deoxycholate amphotericin B formulations for prevention of invasive fungal infections following lung transplantation: A retrospective study. Transpl. Infect. Dis..

[102] Letsou G.V., Safi H.J., Reardon M.J., Ergenoglu M., Li Z., Klonaris C.N., Baldwin J.C., Gilbert B.E., Waldrep J.C. (1999). Pharmacokinetics of liposomal aerosolized cyclosporine A for pulmonary immunosuppression. Ann. Thorac. Surg..

[103] Tagami T., Ando Y., Ozeki T. (2017). Fabrication of liposomal doxorubicin exhibiting ultrasensitivity against phospholipase A2 for efficient pulmonary drug delivery to lung cancers. Int. J. Pharm..

[104] LoPresti S.T., Arral M.L., Chaudhary N., Whitehead K.A. (2022). The replacement of helper lipids with charged alternatives in lipid nanoparticles facilitates targeted mRNA delivery to the spleen and lungs. J. Control. Release.

[105] Garbuzenko O.B., Saad M., Betigeri S., Zhang M., Vetcher A.A., Soldatenkov V.A., Reimer D.C., Pozharov V.P., Minko T. (2009). Intratracheal versus intravenous liposomal delivery of siRNA, antisense oligonucleotides and anticancer drug. Pharm. Res..

[106] Goldstein D.A., Tan Y.K., Soldin S.J. (1987). Pharmacokinetics and absolute bioavailability of salbutamol in healthy adult volunteers. Eur. J. Clin. Pharmacol..

[107] De Leo V., Ruscigno S., Trapani A., Di Gioia S., Milano F., Mandracchia D., Comparelli R., Castellani S., Agostiano A., Trapani G. (2018). Preparation of drug-loaded small unilamellar liposomes and evaluation of their potential for the treatment of chronic respiratory diseases. Int. J. Pharm..

[108] Hickey J.W., Santos J.L., Williford J.M., Mao H.Q. (2015). Control of polymeric nanoparticle size to improve therapeutic delivery. J. Control Release.

[109] Begines B., Ortiz T., Pérez-Aranda M., Martínez G., Merinero M., Argüelles-Arias F., Alcudia A. (2020). Polymeric Nanoparticles for Drug Delivery: Recent Developments and Future Prospects. Nanomaterials.

[110] Gutierrez Cisneros C., Bloemen V., Mignon A. (2021). Synthetic, Natural, and Semisynthetic Polymer Carriers for Controlled Nitric Oxide Release in Dermal Applications: A Review. Polymers.

[111] Li C., Li J., Bai Y., Zhang K., Wang Z., Zhang Y., Guan Q., Wang S., Li Z., Li Z. (2025). Polysialic acid-based nanoparticles for enhanced targeting and controlled dexamethasone release in pulmonary inflammation treatment. Int. J. Biol. Macromol..

[112] Ghazanfary S., Rahmanian M., Vatanchian M., Haghbin A., Shakeri F., Oroojalian F. (2024). Characterization and Efficacy Evaluation of mPEG-PLGA/Taraxasterol Acetate Nanoparticles as Nano-Therapeutic Agents in Asthma Management. BioNanoScience.

[113] Anselmo A.C., Mitragotri S. (2015). A Review of Clinical Translation of Inorganic Nanoparticles. Aaps J..

[114] D’Souza A., Shegokar R., Holban A.M., Grumezescu A.M. (2016). 16—Polymer: Lipid Hybrid Nanostructures in Cancer Drug Delivery: Successes and Limitations. Nanoarchitectonics for Smart Delivery and Drug Targeting.

[115] Feng Q.L., Wu J., Chen G.Q., Cui F.Z., Kim T.N., Kim J.O. (2000). A mechanistic study of the antibacterial effect of silver ions on Escherichia coli and Staphylococcus aureus. J. Biomed Mater. Res..

[116] Martín-Faivre L., Prince L., Cornu C., Villeret B., Sanchez-Guzman D., Rouzet F., Sallenave J.-M., Garcia-Verdugo I. (2025). Pulmonary delivery of silver nanoparticles prevents influenza infection by recruiting and activating lymphoid cells. Biomaterials.

[117] Srinivas A., Rao P.J., Selvam G., Goparaju A., Murthy B.P., Reddy N.P. (2012). Oxidative stress and inflammatory responses of rat following acute inhalation exposure to iron oxide nanoparticles. Hum. Exp. Toxicol..

[118] Yue L., Qidian L., Jiawei W., Rou X., Miao H. (2022). Acute iron oxide nanoparticles exposure induced murine eosinophilic airway inflammation via TLR2 and TLR4 signaling. Environ. Toxicol..

[119] Al Faraj A., Shaik A.S., Afzal S., Al Sayed B., Halwani R. (2014). MR imaging and targeting of a specific alveolar macrophage subpopulation in LPS-induced COPD animal model using antibody-conjugated magnetic nanoparticles. Int. J. Nanomed..

[120] Covarrubias-Zambrano O., Motamedi M., Ameredes B.T., Tian B., Calhoun W.J., Zhao Y., Brasier A.R., Kalubowilage M., Malalasekera A.P., Yapa A.S. (2022). Optical biosensing of markers of mucosal inflammation. Nanomedicine.

[121] Chakraborty A., Pinar A.A., Lam M., Bourke J.E., Royce S.G., Selomulya C., Samuel C.S. (2021). Pulmonary myeloid cell uptake of biodegradable nanoparticles conjugated with an anti-fibrotic agent provides a novel strategy for treating chronic allergic airways disease. Biomaterials.

[122] Jadhav K., Jhilta A., Singh R., Ray E., Sharma N., Shukla R., Singh A.K., Verma R.K. (2023). Clofazimine nanoclusters show high efficacy in experimental TB with amelioration in paradoxical lung inflammation. Biomater. Adv..

[123] Dacco V., Alicandro G., Consales A., Rosazza C., Sciarrabba C.S., Cariani L., Colombo C. (2023). Cepacia syndrome in cystic fibrosis: A systematic review of the literature and possible new perspectives in treatment. Pediatr. Pulmonol..

[124] Costabile G., Provenzano R., Azzalin A., Scoffone V.C., Chiarelli L.R., Rondelli V., Grillo I., Zinn T., Lepioshkin A., Savina S. (2020). PEGylated mucus-penetrating nanocrystals for lung delivery of a new FtsZ inhibitor against Burkholderia cenocepacia infection. Nanomed. Nanotechnol. Biol. Med..

[125] Reddy K.T.K., Madhavi Latha B., Gupta J.K., Chaitanya A., Srinivasa Babu P., Vamseekrishna G., Manikanta Y., Sahithi A., Thomas S., Das P., Ganguly S. (2024). Discovery and History of Quantum Dots. Quantum Dots Based Nanocomposites: Design, Fabrication and Emerging Applications.

[126] Ren L., Wang L., Rehberg M., Stoeger T., Zhang J., Chen S. (2022). Applications and immunological effects of quantum dots on respiratory system. Front. Immunol..

[127] Roberts J.R., Antonini J.M., Porter D.W., Chapman R.S., Scabilloni J.F., Young S.-H., Schwegler-Berry D., Castranova V., Mercer R.R. (2013). Lung toxicity and biodistribution of Cd/Se-ZnS quantum dots with different surface functional groups after pulmonary exposure in rats. Part. Fibre Toxicol..

[128] Lee V., McMahan R.S., Hu X., Gao X., Faustman E.M., Griffith W.C., Kavanagh T.J., Eaton D.L., McGuire J.K., Parks W.C. (2015). Amphiphilic polymer-coated CdSe/ZnS quantum dots induce pro-inflammatory cytokine expression in mouse lung epithelial cells and macrophages. Nanotoxicology.

[129] Ju Y., Hu Y., Yang P., Xie X., Fang B. (2023). Extracellular vesicle-loaded hydrogels for tissue repair and regeneration. Mater. Today Bio.

[130] Hough K.P., Chanda D., Duncan S.R., Thannickal V.J., Deshane J.S. (2017). Exosomes in immunoregulation of chronic lung diseases. Allergy.

[131] Han Y., Zhu Y., Youngblood H.A., Almuntashiri S., Jones T.W., Wang X., Liu Y., Somanath P.R., Zhang D. (2022). Nebulization of extracellular vesicles: A promising small RNA delivery approach for lung diseases. J. Control. Release.

[132] Popowski K.D., Moatti A., Scull G., Silkstone D., Lutz H., de Juan Abad B.L., George A., Belcher E., Zhu D., Mei X. (2022). Inhalable dry powder mRNA vaccines based on extracellular vesicles. Matter.

[133] Kim G., Lee Y., Ha J., Han S., Lee M. (2021). Engineering exosomes for pulmonary delivery of peptides and drugs to inflammatory lung cells by inhalation. J. Control. Release.

[134] Nasr M., Nawaz S., Elhissi A. (2012). Amphotericin B lipid nanoemulsion aerosols for targeting peripheral respiratory airways via nebulization. Int. J. Pharm..

[135] Chen Y.-B., Zhang Y.-B., Wang Y.-L., Kaur P., Yang B.-G., Zhu Y., Ye L., Cui Y.-L. (2022). A novel inhalable quercetin-alginate nanogel as a promising therapy for acute lung injury. J. Nanobiotechnol..

[136] Nasr M., Najlah M., D’Emanuele A., Elhissi A. (2014). PAMAM dendrimers as aerosol drug nanocarriers for pulmonary delivery via nebulization. Int. J. Pharm..

[137] Zakaria M.Y., Abd El-Halim S.M., Beshay B.Y., Zaki I., Abourehab M.A.S. (2023). ‘Poly phenolic phytoceutical loaded nano-bilosomes for enhanced caco-2 cell permeability and SARS-CoV 2 antiviral activity’: In-vitro and insilico studies. Drug Deliv..

[138] Wang Z., Popowski K.D., Zhu D., de Juan Abad B.L., Wang X., Liu M., Lutz H., De Naeyer N., DeMarco C.T., Denny T.N. (2022). Exosomes decorated with a recombinant SARS-CoV-2 receptor-binding domain as an inhalable COVID-19 vaccine. Nat. Biomed. Eng..

[139] Ramachandran S., Prakash P., Mohtar N., Kumar K.S., Parumasivam T. (2023). Review of inhalable nanoparticles for the pulmonary delivery of anti-tuberculosis drugs. Pharm. Dev. Technol..

[140] Geller D., Crowley C., Froehlich J., Schwabe C., O’Carroll M. (2024). WS10. 03 Inhaled LUNAR^®^-CFTR mRNA (ARCT-032) is safe and well-tolerated: A phase 1 study. J. Cyst. Fibros..

[141] Bruns I.B., Fitzgerald M.F., Pardali K., Gardiner P., Keeling D.J., Axelsson L.T., Jiang F., Lickliter J., Close D.R. (2019). Phase 1 evaluation of the inhaled IL-4Rα antagonist, AZD1402/PRS-060, a potent and selective blocker of IL-4Rα. Eur. Respir. J..

[142] U.S. National Library of Medicine https://clinicaltrials.gov/.

[143] Food and Drug Administration (2018). FDA Approves a New Antibacterial Drug to Treat a Serious Lung Disease Using a Novel Pathway to Spur innovation.

[144] Zhu M., Ma L., Zhong P., Huang J., Gai J., Li G., Li Y., Qiao P., Gu H., Li X. (2024). A novel inhalable nanobody targeting IL-4Rα for the treatment of asthma. J. Allergy Clin. Immunol..

[145] Bilton D., Pressler T., Fajac I., Clancy J.P., Sands D., Minic P., Cipolli M., Galeva I., Solé A., Quittner A.L. (2020). Amikacin liposome inhalation suspension for chronic Pseudomonas aeruginosa infection in cystic fibrosis. J. Cyst. Fibros..

[146] Bilton D., Fajac I., Pressler T., Clancy J.P., Sands D., Minic P., Cipolli M., Galeva I., Solé A., Quittner A.L. (2021). Long-term amikacin liposome inhalation suspension in cystic fibrosis patients with chronic P. aeruginosa infection. J. Cyst. Fibros..

[147] Matschiner G., Fitzgerald M.F., Moebius U., Hohlbaum A.M., Gille H., Jensen K., Kirchfeld K., Rattenstetter B., Laforge A., Bel Aiba R.S. (2023). Elarekibep (PRS-060/AZD1402), a new class of inhaled Anticalin medicine targeting IL-4Ra for type 2 endotype asthma. J. Allergy Clin. Immunol..

[148] Rowe S.M., Zuckerman J.B., Dorgan D., Lascano J., McCoy K., Jain M., Schechter M.S., Lommatzsch S., Indihar V., Lechtzin N. (2023). Inhaled mRNA therapy for treatment of cystic fibrosis: Interim results of a randomized, double-blind, placebo-controlled phase 1/2 clinical study. J. Cyst. Fibros..

[149] Alton E.W.F.W., Armstrong D.K., Ashby D., Bayfield K.J., Bilton D., Bloomfield E.V., Boyd A.C., Brand J., Buchan R., Calcedo R. (2016). A Randomised, Double-Blind, Placebo-Controlled Trial of Repeated Nebulisation of Non-Viral Cystic Fibrosis Transmembrane Conductance Regulator (CFTR) Gene Therapy in Patients with Cystic Fibrosis.

[150] Griffith D.E., Eagle G., Thomson R., Aksamit T.R., Hasegawa N., Morimoto K., Addrizzo-Harris D.J., O’Donnell A.E., Marras T.K., Flume P.A. (2018). Amikacin Liposome Inhalation Suspension for Treatment-Refractory Lung Disease Caused by Mycobacterium avium Complex (CONVERT). A Prospective, Open-Label, Randomized Study. Am. J. Respir. Crit. Care Med..

[151] Tyumina O. Evaluation of Safety and Efficiency of Method of Exosome Inhalation in SARS-CoV-2 Associated Pneumonia.(COVID-19EXO). [2021 Mar 10]. ClinicalTrials. gov [Internet]. Samara: US National Library of Medicine. https://clinicaltrials.gov/ct2/show/results/NCT04491240.

[152] Fattal E., Grabowski N., Mura S., Vergnaud J., Tsapis N., Hillaireau H. (2014). Lung toxicity of biodegradable nanoparticles. J. Biomed. Nanotechnol..

[153] Card J.W., Zeldin D.C., Bonner J.C., Nestmann E.R. (2008). Pulmonary applications and toxicity of engineered nanoparticles. Am. J. Physiol. -Lung Cell. Mol. Physiol..

[154] Soto K.F., Murr L.E., Garza K.M. (2008). Cytotoxic Responses and Potential Respiratory Health Effects of Carbon and Carbonaceous Nanoparticulates in the Paso del Norte Airshed Environment. Int. J. Environ. Res. Public Health.

[155] Wu T., Tang M. (2014). Toxicity of quantum dots on respiratory system. Inhal. Toxicol..

[156] Lahouty M., Fadaee M., Shanehbandi D., Kazemi T. (2024). Exosome-driven nano-immunotherapy: Revolutionizing colorectal cancer treatment. Mol. Biol. Rep..

[157] Vega-Villa K.R., Takemoto J.K., Yáñez J.A., Remsberg C.M., Forrest M.L., Davies N.M. (2008). Clinical toxicities of nanocarrier systems. Adv. Drug Deliv. Rev..

[158] Lee S.-H., Wang T.-Y., Hong J.-H., Cheng T.-J., Lin C.-Y. (2016). NMR-based metabolomics to determine acute inhalation effects of nano-and fine-sized ZnO particles in the rat lung. Nanotoxicology.

[159] Dobson J. (2007). Toxicological aspects and applications of nanoparticles in paediatric respiratory disease. Paediatr. Respir. Rev..

[160] Huh D., Matthews B.D., Mammoto A., Montoya-Zavala M., Hsin H.Y., Ingber D.E. (2010). Reconstituting organ-level lung functions on a chip. Science.

[161] Chan H.W., Chow S., Zhang X., Zhao Y., Tong H.H.Y., Chow S.F. (2023). Inhalable Nanoparticle-based Dry Powder Formulations for Respiratory Diseases: Challenges and Strategies for Translational Research. AAPS PharmSciTech.

[162] Murday J.S., Siegel R.W., Stein J., Wright J.F. (2009). Translational nanomedicine: Status assessment and opportunities. Nanomed. Nanotechnol. Biol. Med..

[163] Feng J., Markwalter C.E., Tian C., Armstrong M., Prud’homme R.K. (2019). Translational formulation of nanoparticle therapeutics from laboratory discovery to clinical scale. J. Transl. Med..

[164] U.S. Food and Drug Administration (2022). Drug Products, Including Biological Products, That Contain Nanomaterials: Guidance for Industry.

[165] Paliwal R., Babu R.J., Palakurthi S. (2014). Nanomedicine scale-up technologies: Feasibilities and challenges. AAPS PharmSciTech.

[166] Khairnar S.V., Pagare P., Thakre A., Nambiar A.R., Junnuthula V., Abraham M.C., Kolimi P., Nyavanandi D., Dyawanapelly S. (2022). Review on the Scale-Up Methods for the Preparation of Solid Lipid Nanoparticles. Pharmaceutics.

[167] Klinkova A., Thérien-Aubin H., Klinkova A., Thérien-Aubin H. (2024). Chapter 3—Inorganic nanoparticles. Nanochemistry.

[168] Junghanns J.U., Müller R.H. (2008). Nanocrystal technology, drug delivery and clinical applications. Int. J. Nanomed..

[169] Pielenhofer J., Meiser S.L., Gogoll K., Ciciliani A.-M., Denny M., Klak M., Lang B.M., Staubach P., Grabbe S., Schild H. (2023). Quality by Design (QbD) Approach for a Nanoparticulate Imiquimod Formulation as an Investigational Medicinal Product. Pharmaceutics.

[170] Azad M.A., Capellades G., Wang A.B., Klee D.M., Hammersmith G., Rapp K., Brancazio D., Myerson A.S. (2021). Impact of Critical Material Attributes (CMAs)-Particle Shape on Miniature Pharmaceutical Unit Operations. AAPS PharmSciTech.

[171] Hu F., Ma S., Hu T. (2025). Mechanistic Analysis of Fluid Dynamics and Multifactorial Impact Mechanisms in Inhaled Pharmaceutical Deposition for Chronic Respiratory Diseases. Bioengineering.

[172] Heredero J., Peña Á., Broset E., Blandín B., de Miguel D., Alejo T., Toro A., Mata E., López-Gavín A., Gallego-Lleyda A. (2025). Predictive Lung-and Spleen-Targeted mRNA Delivery with Biodegradable Ionizable Lipids in Four-Component LNPs. Pharmaceutics.

[173] Hou X., Zaks T., Langer R., Dong Y. (2021). Lipid nanoparticles for mRNA delivery. Nat. Rev. Mater..

[174] Foster J.M., Usherwood T., Smith L., Sawyer S.M., Xuan W., Rand C.S., Reddel H.K. (2014). Inhaler reminders improve adherence with controller treatment in primary care patients with asthma. J. Allergy Clin. Immunol..

[175] Chan A.H., Stewart A.W., Harrison J., Camargo C.A., Black P.N., Mitchell E.A. (2015). The effect of an electronic monitoring device with audiovisual reminder function on adherence to inhaled corticosteroids and school attendance in children with asthma: A randomised controlled trial. Lancet Respir. Med..

[176] Moon C., Smyth H.D.C., Watts A.B., Williams R.O. (2019). Delivery Technologies for Orally Inhaled Products: An Update. AAPS PharmSciTech.

[177] Chiang C.-E., Wu J.-C., Lin H.-L., Chiu L.-C., Liu C.-T., Liu Y.-F., Jhunjhunwala M., Chen C.-S. (2024). Late Breaking Abstract - Engineering an AI-base mesh nebulizer system for personalized respiratory drug delivery. Eur. Respir. J..

[178] Islam M.R., Liu C., Cai C., Shah J., Feng Y. (2024). A user-centered smart inhaler algorithm for targeted drug delivery in juvenile onset recurrent respiratory papillomatosis treatment integrating computational fluid particle dynamics and machine learning. Phys. Fluids.

[179] Bannuscher A., Karkossa I., Buhs S., Nollau P., Kettler K., Balas M., Dinischiotu A., Hellack B., Wiemann M., Luch A. (2020). A multi-omics approach reveals mechanisms of nanomaterial toxicity and structure–activity relationships in alveolar macrophages. Nanotoxicology.

[180] Forest V., Pourchez J. (2022). Nano-delivery to the lung-by inhalation or other routes and why nano when micro is largely sufficient?. Adv. Drug Deliv. Rev..

[181] Khatib I., Khanal D., Ruan J., Cipolla D., Dayton F., Blanchard J.D., Chan H.-K. (2019). Ciprofloxacin nanocrystals liposomal powders for controlled drug release via inhalation. Int. J. Pharm..

[182] Beck-Broichsitter M., Knuedeler M.-C., Oesterheld N., Seeger W., Schmehl T. (2014). Boosting the aerodynamic properties of vibrating-mesh nebulized polymeric nanosuspensions. Int. J. Pharm..

[183] Dailey L.A., Schmehl T., Gessler T., Wittmar M., Grimminger F., Seeger W., Kissel T. (2003). Nebulization of biodegradable nanoparticles: Impact of nebulizer technology and nanoparticle characteristics on aerosol features. J. Control. Release.

[184] Beck-Broichsitter M., Kleimann P., Gessler T., Seeger W., Kissel T., Schmehl T. (2012). Nebulization performance of biodegradable sildenafil-loaded nanoparticles using the Aeroneb^®^ Pro: Formulation aspects and nanoparticle stability to nebulization. Int. J. Pharm..

[185] Rogueda P.G., Traini D. (2007). The nanoscale in pulmonary delivery. Part 2: Formulation platforms. Expert Opin. Drug Deliv..

[186] Mangal S., Gao W., Li T., Zhou Q. (2017). Pulmonary delivery of nanoparticle chemotherapy for the treatment of lung cancers: Challenges and opportunities. Acta Pharmacol. Sin..

[187] Tan Y., Yang Z., Peng X., Xin F., Xu Y., Feng M., Zhao C., Hu H., Wu C. (2011). A novel bottom-up process to produce nanoparticles containing protein and peptide for suspension in hydrofluoroalkane propellants. Int. J. Pharm..

[188] Sharma K., Somavarapu S., Colombani A., Govind N., Taylor K.M.G. (2012). Crosslinked chitosan nanoparticle formulations for delivery from pressurized metered dose inhalers. Eur. J. Pharm. Biopharm..

[189] Vallorz E., Sheth P., Myrdal P. (2019). Pressurized Metered Dose Inhaler Technology: Manufacturing. AAPS PharmSciTech.

[190] Sheth P., Grimes M.R., Stein S.W., Myrdal P.B. (2017). Impact of droplet evaporation rate on resulting in vitro performance parameters of pressurized metered dose inhalers. Int. J. Pharm..

[191] Kumar R., Mehta P., Shankar K.R., Rajora M.A.K., Mishra Y.K., Mostafavi E., Kaushik A. (2022). Nanotechnology-Assisted Metered-Dose Inhalers (MDIs) for High-Performance Pulmonary Drug Delivery Applications. Pharm. Res..

[192] Thorat S., Meshram S. (2015). Formulation and product development of pressurised metered dose inhaler: An overview. PharmaTutor.

[193] Shiehzadeh F., Tafaghodi M. (2016). Dry powder form of polymeric nanoparticles for pulmonary drug delivery. Curr. Pharm. Des..

[194] Yeganeh E.M., Bagheri H., Mahjub R. (2020). Preparation, statistical optimization and in-vitro characterization of a dry powder inhaler (DPI) containing solid lipid nanoparticles encapsulating amphotericin B: Ion paired complexes with distearoyl phosphatidylglycerol. Iran. J. Pharm. Res. IJPR.

[195] Topal G.R., Devrim B., Eryilmaz M., Bozkir A. (2018). Design of ciprofloxacin-loaded nano-and microcomposite particles for dry powder inhaler formulations: Preparation, in vitro characterisation, and antimicrobial efficacy. J. Microencapsul..

[196] Mehta P. (2016). Dry Powder Inhalers: A Focus on Advancements in Novel Drug Delivery Systems. J. Drug Deliv..

[197] Dahmash E.Z., Achkar N.R., Ali D.K., Jarrar Q., Iyire A., Assaf S.M., Alyami H. (2024). Preclinical evaluation of novel synthesised nanoparticles based on tyrosine poly(ester amide) for improved targeted pulmonary delivery. Sci. Rep..

[198] Hejduk A., Urbańska A., Osiński A., Łukaszewicz P., Domański M., Sosnowski T.R. (2018). Technical challenges in obtaining an optimized powder/DPI combination for inhalation delivery of a bi-component generic drug. J. Drug Deliv. Sci. Technol..

[199] Hirota K., Terada H., Ohshima H., Makino K. (2014). Chapter 5—Particle-manufacturing technology-based inhalation therapy for pulmonary diseases. Colloid and Interface Science in Pharmaceutical Research and Development.

[200] Chan H.-K., Chew N.Y.K. (2003). Novel alternative methods for the delivery of drugs for the treatment of asthma. Adv. Drug Deliv. Rev..

[201] Wanning S., Süverkrüp R., Lamprecht A. (2015). Pharmaceutical spray freeze drying. Int. J. Pharm..

[202] Vass P., Démuth B., Hirsch E., Nagy B., Andersen S.K., Vigh T., Verreck G., Csontos I., Nagy Z.K., Marosi G. (2019). Drying technology strategies for colon-targeted oral delivery of biopharmaceuticals. J. Control. Release.

[203] Liang W., Pan H.W., Vllasaliu D., Lam J.K.W. (2020). Pulmonary Delivery of Biological Drugs. Pharmaceutics.

[204] Movellan J., Murgia X., Gracia R., Marradi M., Miranda J.I., Aizpurua J.M., Grande H.-J., Dupin D., Loinaz I. (2025). Tobramycin nanoformulation for chronic pulmonary infections: From drug product definition to scale-up for preclinical evaluation. Int. J. Pharm..

[205] Yeo L.Y., Friend J.R., McIntosh M.P., Meeusen E.N., Morton D.A. (2010). Ultrasonic nebulization platforms for pulmonary drug delivery. Expert Opin. Drug Deliv..

[206] Metz J.K., Scharnowske L., Hans F., Schnur S., Knoth K., Zimmer H., Limberger M., Groß H., Lehr C.-M., Hittinger M. (2020). Safety assessment of excipients (SAFE) for orally inhaled drug products. ALTEX-Altern. Anim. Exp..

[207] Zhang J., Wu L., Chan H.-K., Watanabe W. (2011). Formation, characterization, and fate of inhaled drug nanoparticles. Adv. Drug Deliv. Rev..

[208] Gonsalves A., Menon J.U. (2024). Impact of Nebulization on the Physicochemical Properties of Polymer–Lipid Hybrid Nanoparticles for Pulmonary Drug Delivery. Int. J. Mol. Sci..

[209] de Pablo E., Fernández-García R., Ballesteros M.P., Torrado J.J., Serrano D.R. (2017). Nebulised antibiotherapy: Conventional versus nanotechnology-based approaches, is targeting at a nano scale a difficult subject?. Ann. Transl. Med..

[210] Zaru M., Mourtas S., Klepetsanis P., Fadda A.M., Antimisiaris S.G. (2007). Liposomes for drug delivery to the lungs by nebulization. Eur. J. Pharm. Biopharm..

[211] Choi W.S., Murthy G.K., Edwards D.A., Langer R., Klibanov A.M. (2001). Inhalation delivery of proteins from ethanol suspensions. Proc. Natl. Acad. Sci..

[212] Ghanem R., Buin X., Haute T., Philippe J., Kaouane G., Leclerc L., Guivarch M., Le Gall T., Pourchez J., Montier T. (2025). Impact of nebulizers on nanoparticles-based gene delivery efficiency: In vitro and in vivo comparison of jet and mesh nebulizers using branched-polyethyleneimine. Drug Deliv..

[213] Fu T.-T., Cong Z.-Q., Zhao Y., Chen W.-Y., Liu C.-Y., Zheng Y., Yang F.-F., Liao Y.-H. (2019). Fluticasone propionate nanosuspensions for sustained nebulization delivery: An in vitro and in vivo evaluation. Int. J. Pharm..

[214] Makled S., Nafee N., Boraie N. (2017). Nebulized solid lipid nanoparticles for the potential treatment of pulmonary hypertension via targeted delivery of phosphodiesterase-5-inhibitor. Int. J. Pharm..

[215] Beck-Broichsitter M., Kleimann P., Schmehl T., Betz T., Bakowsky U., Kissel T., Seeger W. (2012). Impact of lyoprotectants for the stabilization of biodegradable nanoparticles on the performance of air-jet, ultrasonic, and vibrating-mesh nebulizers. Eur. J. Pharm. Biopharm..

[216] Lavorini F., Buttini F., Usmani O.S., Barrett J.E., Page C.P., Michel M.C. (2019). 100 Years of Drug Delivery to the Lungs. Concepts and Principles of Pharmacology: 100 Years of the Handbook of Experimental Pharmacology.

[217] (2021). Klein DM, Poortinga A, Verhoeven FM, Bonn D, Bonnet S, van Rijn CJM. Degradation of lipid-based drug delivery formulations during nebulization. Chem Phys..

[218] Lin H.-L., Chen C.-S., Fink J.B., Lee G.-H., Huang C.-W., Chen J.-C., Chiang Z.Y. (2020). In vitro evaluation of a vibrating-mesh nebulizer repeatedly use over 28 days. Pharmaceutics.

[219] Steckel H., Eskandar F. (2003). Factors affecting aerosol performance during nebulization with jet and ultrasonic nebulizers. Eur. J. Pharm. Sci..

[220] Stabile S.G.G., Perez N., Jerez H.E., Simioni Y.R., Butassi E., Mizrahi M.D., Nobile M.L., Perez A.P., Morilla M.J., Higa L.H. (2025). Nebulized Hybrid Nanoarchaeosomes: Anti-Inflammatory Activity, Anti-Microbial Activity and Cytotoxicity on A549 Cells. Int. J. Mol. Sci..

[221] Patel A.K., Kaczmarek J.C., Bose S., Kauffman K.J., Mir F., Heartlein M.W., DeRosa F., Langer R., Anderson D.G. (2019). Inhaled nanoformulated mRNA polyplexes for protein production in lung epithelium. Adv. Mater..

[222] Mišík O., Kejíková J., Cejpek O., Malý M., Jugl A., Bělka M., Mravec F., Lízal F. (2024). Nebulization and In Vitro Upper Airway Deposition of Liposomal Carrier Systems. Mol. Pharm..

[223] Beck-Broichsitter M., Knuedeler M.-C., Schmehl T., Seeger W. (2013). Following the Concentration of Polymeric Nanoparticles During Nebulization. Pharm. Res..

[224] Chauhan G., Wang X., Quadros M., Vats M., Gupta V. (2024). Chitosan/bovine serum albumin layer-by-layer assembled particles for non-invasive inhaled drug delivery to the lungs. Int. J. Biol. Macromol..

[225] Anderson D.S., Patchin E.S., Silva R.M., Uyeminami D.L., Sharmah A., Guo T., Das G.K., Brown J.M., Shannahan J., Gordon T. (2015). Influence of Particle Size on Persistence and Clearance of Aerosolized Silver Nanoparticles in the Rat Lung. Toxicol. Sci..

[226] Wiedmann T.S., DeCastro L., Wood R.W. (1997). Nebulization of NanoCrystals(TM): Production of a Respirable Solid-in-Liquid-in-Air Colloidal Dispersion. Pharm. Res..

[227] Elhissi A., Hidayat K., Phoenix D.A., Mwesigwa E., Crean S., Ahmed W., Faheem A., Taylor K.M.G. (2013). Air-jet and vibrating-mesh nebulization of niosomes generated using a particulate-based proniosome technology. Int. J. Pharm..

[228] Popowski K.D., López de Juan Abad B., George A., Silkstone D., Belcher E., Chung J., Ghodsi A., Lutz H., Davenport J., Flanagan M. (2022). Inhalable exosomes outperform liposomes as mRNA and protein drug carriers to the lung. Extracell. Vesicle.

[229] Amani A., York P., Chrystyn H., Clark B.J. (2010). Evaluation of a Nanoemulsion-Based Formulation for Respiratory Delivery of Budesonide by Nebulizers. AAPS PharmSciTech.

[230] Khan I., Lau K., Bnyan R., Houacine C., Roberts M., Isreb A., Elhissi A., Yousaf S. (2020). A Facile and Novel Approach to Manufacture Paclitaxel-Loaded Proliposome Tablet Formulations of Micro or Nano Vesicles for Nebulization. Pharm. Res..

[231] Rao L., Zhu P., Guo M., Hu M., Guo X., Du Y., Xu G. (2024). Nebulized inhalation of nintedanib-loaded biomimetic nano-liposomes attenuated bleomycin-induced interstitial lung fibrosis in mice. Nano Today.

[232] Abdellatif A.A.H., Khan R.A., Alhowail A.H., Alqasoumi A., Sajid S.M., Mohammed A.M., Alsharidah M., Rugaie O.A., Mousa A.M. (2022). Octreotide-conjugated silver nanoparticles for active targeting of somatostatin receptors and their application in a nebulized rat model. Nanotechnol. Rev..

[233] Ostrander K.D., Bosch H.W., Bondanza D.M. (1999). An in-vitro assessment of a NanoCrystal™ beclomethasone dipropionate colloidal dispersion via ultrasonic nebulization. Eur. J. Pharm. Biopharm..

[234] Zhao D., Li D., Cheng X., Zou Z., Chen X., He C. (2022). Mucoadhesive, Antibacterial, and Reductive Nanogels as a Mucolytic Agent for Efficient Nebulized Therapy to Combat Allergic Asthma. ACS Nano.

[235] Lee J., Han C.H., Oh I.H., Allu S., Kim H.J., Kim J., Kim W.-S., Park B.J. (2024). Fabrication and evaluation of stable amorphous polymer-drug composite particles via a nozzle-free ultrasonic nebulizer. Int. J. Pharm..

[236] Sinswat P., Overhoff K.A., McConville J.T., Johnston K.P., Williams R.O. (2008). Nebulization of nanoparticulate amorphous or crystalline tacrolimus—Single-dose pharmacokinetics study in mice. Eur. J. Pharm. Biopharm..

[237] Kleemann E., Schmehl T., Gessler T., Bakowsky U., Kissel T., Seeger W. (2007). Iloprost-Containing Liposomes for Aerosol Application in Pulmonary Arterial Hypertension: Formulation Aspects and Stability. Pharm. Res..

[238] Elhissi A., Faizi M., Naji W., Gill H., Taylor K. (2007). Physical stability and aerosol properties of liposomes delivered using an air-jet nebulizer and a novel micropump device with large mesh apertures. Int. J. Pharm..

[239] Müller J.T., Kromer A.P.E., Ezaddoustdar A., Alexopoulos I., Steinegger K.M., Porras-Gonzalez D.L., Berninghausen O., Beckmann R., Braubach P., Burgstaller G. (2025). Nebulization of RNA-Loaded Micelle-Embedded Polyplexes as a Potential Treatment of Idiopathic Pulmonary Fibrosis. ACS Appl. Mater. Interfaces.

[240] Naveen K., Bose S., Basheer C., Zare R.N., Gnanamani E. (2024). Handheld portable device for delivering capped silver nanoparticles for antimicrobial applications. QRB Discov..

[241] Zhu Y.-G., Shi M.-m., Monsel A., Dai C.-x., Dong X., Shen H., Li S.-k., Chang J., Xu C.-l., Li P. (2022). Nebulized exosomes derived from allogenic adipose tissue mesenchymal stromal cells in patients with severe COVID-19: A pilot study. Stem Cell Res. Ther..

[242] Merckx P., Lammens J., Nuytten G., Bogaert B., Guagliardo R., Maes T., Vervaet C., De Beer T., De Smedt S.C., Raemdonck K. (2020). Lyophilization and nebulization of pulmonary surfactant-coated nanogels for siRNA inhalation therapy. Eur. J. Pharm. Biopharm..

[243] Unsworth C., Dwyer A.B., Savage A.C., Hobson J.J., Massam J., McDonald T.O., Curley P., Owen A., O’Sullivan A., MacLoughlin R. (2025). Development of solid drug nanoparticle dispersions for pulmonary delivery of niclosamide and nitazoxanide via vibrating mesh nebulisation. RSC Pharm..

[244] Debnath S.K., Saisivam S., Omri A. (2017). PLGA Ethionamide Nanoparticles for Pulmonary Delivery: Development and in vivo evaluation of dry powder inhaler. J. Pharm. Biomed. Anal..

[245] Arora S., Haghi M., Loo C.-Y., Traini D., Young P.M., Jain S. (2015). Development of an Inhaled Controlled Release Voriconazole Dry Powder Formulation for the Treatment of Respiratory Fungal Infection. Mol. Pharm..

[246] Shah S.P., Misra A. (2004). Liposomal amikacin dry powder inhaler: Effect of fines on in vitro performance. AAPS PharmSciTech.

[247] Joshi M.R., Misra A. (2015). Liposomal budesonide for dry powder inhaler: Preparation and stabilization. AAPS PharmSciTech.

[248] AboulFotouh K., Almanza G., Yu Y.-S., Joyce R., Davenport G.J., Cano C., Williams Iii R.O., Zanetti M., Cui Z. (2024). Inhalable dry powders of microRNA-laden extracellular vesicles prepared by thin-film freeze-drying. Int. J. Pharm..

[249] Rosière R., Van Woensel M., Mathieu V., Langer I., Mathivet T., Vermeersch M., Amighi K., Wauthoz N. (2016). Development and evaluation of well-tolerated and tumor-penetrating polymeric micelle-based dry powders for inhaled anti-cancer chemotherapy. Int. J. Pharm..

[250] Casula L., Craparo E.F., Lai E., Scialabba C., Valenti D., Schlich M., Sinico C., Cavallaro G., Lai F. (2024). Encapsulation of Nanocrystals in Mannitol-Based Inhalable Microparticles via Spray-Drying: A Promising Strategy for Lung Delivery of Curcumin. Pharmaceuticals.

[251] Malamatari M. (2016). Engineering Nanoparticle Agglomerates as Dry Powders for Pulmonary Drug Delivery.

[252] Bielski E., Zhong Q., Mirza H., Brown M., Molla A., Carvajal T., da Rocha S.R.P. (2017). TPP-dendrimer nanocarriers for siRNA delivery to the pulmonary epithelium and their dry powder and metered-dose inhaler formulations. Int. J. Pharm..

[253] Price D.N., Stromberg L.R., Kunda N.K., Muttil P. (2017). In Vivo Pulmonary Delivery and Magnetic-Targeting of Dry Powder Nano-in-Microparticles. Mol. Pharm..

[254] Huang Y., Chang Z., Gao Y., Ren C., Lin Y., Zhang X., Wu C., Pan X., Huang Z. (2024). Overcoming the Low-Stability Bottleneck in the Clinical Translation of Liposomal Pressurized Metered-Dose Inhalers: A Shell Stabilization Strategy Inspired by Biomineralization. Int. J. Mol. Sci..

[255] Nyambura B.K., Kellaway I.W., Taylor K.M.G. (2009). Insulin nanoparticles: Stability and aerosolization from pressurized metered dose inhalers. Int. J. Pharm..

[256] Li H.-Y., Zhang F., Ferrari E., Soloviev M. (2020). Preparation of Spray-Dried Nanoparticles for Efficient Drug Delivery to the Lungs. Nanoparticles in Biology and Medicine: Methods and Protocols.

[257] Changsan N., Atipairin A., Muenraya P., Sritharadol R., Srichana T., Balekar N., Sawatdee S. (2024). In Vitro Evaluation of Colistin Conjugated with Chitosan-Capped Gold Nanoparticles as a Possible Formulation Applied in a Metered-Dose Inhaler. Antibiotics.

[258] Vehring R., Lechuga-Ballesteros D., Joshi V., Noga B., Dwivedi S.K. (2012). Cosuspensions of Microcrystals and Engineered Microparticles for Uniform and Efficient Delivery of Respiratory Therapeutics from Pressurized Metered Dose Inhalers. Langmuir.

[259] Miao H., Huang K., Li Y., Li R., Zhou X., Shi J., Tong Z., Sun Z., Yu A. (2023). Optimization of formulation and atomization of lipid nanoparticles for the inhalation of mRNA. Int. J. Pharm..

